# Ultra-high thermal effusivity materials for resonant ambient thermal energy harvesting

**DOI:** 10.1038/s41467-018-03029-x

**Published:** 2018-02-14

**Authors:** Anton L. Cottrill, Albert Tianxiang Liu, Yuichiro Kunai, Volodymyr B. Koman, Amir Kaplan, Sayalee G. Mahajan, Pingwei Liu, Aubrey R. Toland, Michael S. Strano

**Affiliations:** Department of Chemical Engineering, Massachusetts Institute of Technology, 77 Massachusetts Ave., Cambridge, MA 02139 USA

## Abstract

Materials science has made progress in maximizing or minimizing the thermal conductivity of materials; however, the thermal effusivity—related to the product of conductivity and capacity—has received limited attention, despite its importance in the coupling of thermal energy to the environment. Herein, we design materials that maximize the thermal effusivity by impregnating copper and nickel foams with conformal, chemical-vapor-deposited graphene and octadecane as a phase change material. These materials are ideal for ambient energy harvesting in the form of what we call thermal resonators to generate persistent electrical power from thermal fluctuations over large ranges of frequencies. Theory and experiment demonstrate that the harvestable power for these devices is proportional to the thermal effusivity of the dominant thermal mass. To illustrate, we measure persistent energy harvesting from diurnal frequencies, extracting as high as 350 mV and 1.3 mW from approximately 10 °C diurnal temperature differences.

## Abstract (web summary)

Ambient environmental thermal fluctuations offer an abundant yet difficult to harvest renewable energy source, when compared to static thermal gradients. Here, by tuning the thermal effusivity of composite phase change materials, the authors are able to harvest energy from diurnal ambient temperature changes.

## Introduction

Thermal effusivity or thermal inertia is the measure of a material’s ability to transfer thermal energy with its surroundings and is equal to the square root of the product of thermal conductivity and heat capacity^1,2^. The importance of high thermal conductivity and/or capacity materials has been acknowledged for applications in electronic circuits^3–5^, off-peak electricity storage^6,7^, energy-saving buildings^7,8^, and chemical reactors^9^. However, to date, the explicit maximization of thermal effusivity has received limited attention in materials science, despite the potential to dynamically capture and store thermal energy from the environment. Examples of materials in the literature with high thermal effusivities include phase change materials (PCMs) within highly thermally conductive matrices^10–12^, high thermal conductivity particles dispersed in PCMs^6,13,14^, and microencapsulated PCMs^6,15^. Hydrated salts have also received attention due to their high heat storage capacity and respectable thermal conductivity^16^. Despite this progress, the development of high thermal effusivity materials remains relatively unexplored, as the above-mentioned studies have mainly focused on enhancing either thermal conductivity or thermal capacitance and neglected the significance of their product.

There is substantial interest in developing renewable energy harvesting technologies^17^. Some of the largest capacity examples of such technologies have intermittency as a primary limitation, including solar and wind^17^. Environmental, thermal energy harvesting can be a potential solution to augment these technologies and also has applications for the remote powering of sensor networks and miniaturized mobile electronics^18,19^. Thermal energy harvesting technologies can be divided into static and transient approaches. Static temperature gradients of various forms are widely utilized in alternative energy technologies, including solar thermal energy harvesting^20^. In addition, thermal energy harvesting with thermoelectric devices, situated in specific locations with spatial temperature gradients, has also been proposed^19^. Nevertheless, thermoelectric energy harvesting from a static gradient is limited by the large thermal conductivities of the constituent materials, which reduce the applied gradient and the conversion efficiency of the device^21^. Thus far, only a few works have considered ambient and generic thermal fluctuations that occur in the environment as a persistent, renewable energy source to augment existing technologies. In particular, pyroelectric energy harvesters^18^ have been developed for harvesting from high-frequency personal temperature variations^21^ and wind-induced temperature variations^22^. Although pyroelectric energy harvesting has been shown to have a higher conversion efficiency compared to static, thermoelectric energy harvesting, the power generated by pyroelectricity, is strongly limited by its reliance on high-frequency temperature variations^18^. Alternatively, transient thermal energy harvesting has been proposed for harvesting energy from the temperature differences between ambient air and large thermal capacitors (e.g., building walls or soil) with thermoelectrics^23–27^. Researchers have also considered power generation from transient temperature changes by tuning heat exchanger parameters, in a lumped analysis considering small Biot numbers, on either side of a thermoelectric^28,29^. It should also be noted that recent efforts have focused on thermoelectric energy harvesting from diurnal^30^ and aircraft-related^31^ temperature changes with the employment of PCMs for latent heat storage. However, these strategies have not been optimized—in terms of material and device designs—to obtain the maximum power generation from generic, broadband, and ambient temperature fluctuations existing in the environment.

Herein, we advance a set of ultra-high thermal effusivity materials, as well as their application to environmental, ambient energy harvesting, using what we term a thermal resonator device. We show that such devices utilize high thermal effusivity materials to optimize their electrical power output from ambient thermal fluctuations. We derive an analytical model for the design of such devices, validate the model experimentally, and reduce to practice a power-optimized device for usable electrical energy generation based on diurnal, ambient temperature fluctuations. Our work is distinct in both the development and experimental demonstration of a thermal resonator, or a self-contained, portable device capable of a broadband input of a range of temperature waveforms for the direct conversion to electrical energy. Our approach fundamentally differs from existing methods of thermal energy harvesting that rely on a static thermal gradient imposed upon a thermoelectric device^19^. In contrast to pyroelectrics, our concept of a thermal resonator has the capability of being tuned for optimal performance at a target frequency of temperature oscillations and is not limited to high-frequency temperature fluctuations. Lastly, our thermal resonance devices—self-contained, portable, and power-optimized to a generic temperature waveform—are distinct from other transient thermal energy harvesting schemes with thermoelectrics^23–31^.

## Results

### Ultra-high thermal effusivity materials

Mathematically, the thermal effusivity, *e*, is related to the product of the thermal conductivity, *k*, and volumetric heat capacity, *C*:^1^

1 $$e = \sqrt {kC} = \sqrt {k\rho C_{\mathrm{p}}} ,$$

where *ρ* is density and *C*_p_ is specific heat.

If a PCM is utilized in a dynamic environment in proximity to its phase transition temperature, an alternate, effective thermal effusivity, *e*_eff,_ should be used instead:

2 $$e_{{\mathrm{eff}}} = \sqrt {k\rho h} ,$$

where *h* is the latent heat per unit mass (Supplementary Note 1, Supplementary Fig. 1). The incorporation of PCMs in proximity to their phase transition temperatures results in an enhancement of approximately an order of magnitude in energy storage per unit volume in comparison to sensible heat storage^7^.

We have prepared two ultra-high thermal effusivity materials through the incorporation of conformal, chemical-vapor-deposited (CVD) multi-layer graphene (G) into PCM composites, which consist of the PCM octadecane (OD) and a copper (Cu) or nickel (Ni) foam (Methods). We label these materials Cu/G/OD and Ni/G/OD, respectively. The metal foam serves as a highly thermally conducting and porous matrix, while the OD bolsters the thermal capacitance through the latent heat of its phase change. OD is chosen as the PCM because its phase transition temperature (27.7 °C) makes it amenable to ambient energy harvesting^6^. The incorporation of carbon nanomaterials, such as graphene, can enhance the thermal conductivity, while negligibly affecting the thermal capacitance because of the low volume fractions needed to affect thermal conduction^13,14^.

**Fig. 1:**
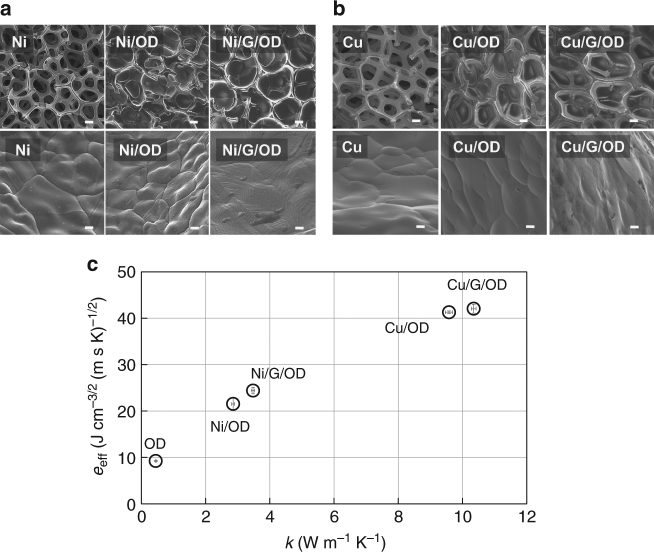
Fabrication and characterization of ultra-high thermal effusivity materials. **a** SEMs for pristine Ni foam (Ni), Ni foam impregnated with OD (Ni/OD), and Ni/G/OD (left to right). Scale bars: top row (100 μm), bottom row (1 μm). **b** SEMs for pristine Cu foam (Cu), Cu foam impregnated with OD (Cu/OD), and Cu/G/OD (left to right). Scale bars: top row (100 μm), bottom row (1 μm). **c** Scatter plot showing the enhancement in effective thermal effusivity for our ultra-high thermal effusivity materials compared with a standard PCM (OD) and its metal foam composites as a function of the thermal conductivity of the composite. Error bars represent 95% confidence intervals

To fabricate Cu/G/OD and Ni/G/OD materials, we grow multi-layer graphene on Cu^32^ and Ni^33^ foams, which are then vacuum impregnated with molten OD (Methods). Scanning electron micrographs (SEMs) for the pristine Ni foam, OD-impregnated Ni foam (Ni/OD), and Ni/G/OD are shown in Fig. 1a. One can clearly identify the grain boundaries of the Ni lattice for the original Ni foam, especially in the magnified micrograph on the bottom. Once infused with OD, one can identify the filled pores, with the detailed Ni grain boundaries remaining unchanged. The multi-layer graphene coating shown in the third column, however, appears to have completely covered the Ni surface, bridging the grain boundaries, as evidenced by the layered fingerprint-type structure shown in the micrograph. This connectivity can improve heat transfer across Ni grain boundaries, as well as augment interfacial heat transfer between the OD and the Ni foam. SEMs for the pristine Cu foam, OD impregnated Cu foam, and Cu/G/OD are also provided in Fig. 1b. Similar interpretations and conclusions can be developed for the Cu foam PCM composites.

**Fig. 2:**
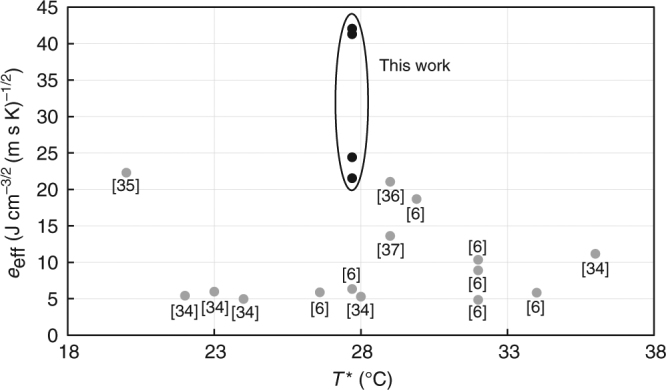
Master plot for effective thermal effusivity. Master plot of effective thermal effusivity for phase change materials measured in the literature and this work (references in brackets^6, 34–37^). Results are confined to isotropic and ambient phase change materials (transition temperature T^*^ occurring between 20 and 40 °C), for which volume-specific latent heat and thermal conductivity are directly or indirectly reported. The four data points enclosed in the ellipse refer to this work for graphene-modified and pristine Ni and Cu foams infused with OD

To further characterize the Ni/G/OD and Cu/G/OD materials, we perform Raman spectroscopy, latent heat, and thermal conductivity measurements. Raman spectra for the metal foams before and after conformal, CVD graphene growth indicate the presence of multi-layer graphene (Supplementary Fig. 2, Supplementary Methods), as evidenced by the intensities of the 2D peaks relative to the G peaks (ratio < 1)^32^. Furthermore, the results of the thermal conductivity and latent heat measurements are used to calculate thermal effusivity, according to Eq. (2) (Methods, Supplementary Notes 2, 3, Supplementary Figs. 3–7). We observe a significant enhancement in the thermal conductivity and effective thermal effusivity of our Ni/G/OD ultra-high thermal effusivity material as compared with Ni/OD (Fig. 1c). Similar enhancements in thermal conductivity and effective thermal effusivity are observed when comparing Cu/G/OD to Cu/OD. Our values of effective thermal effusivity and latent heat agree well with predictions from Hashin–Shtrikman (HS) theory (Supplementary Fig. 8, Supplementary Note 4) and Supplementary Eq. (10), respectively. The latter agreement supports complete OD impregnation of the metal foams. To compare our synthesized materials with the existing literature, we generated a master plot of effective thermal effusivity vs. phase transition temperature, T^*^ for PCMs (Fig. 2)^6,34–37^. The PCMs incorporated into the master plot are ambient (T^*^ between 20 and 40 °C) and isotropic (Supplementary Table 1). As can be seen from the master plot, our ultra-high thermal effusivity materials exceed all values in the literature to date.

The enhancements for conductivity and effusivity are not as drastic for the Cu composites as compared to the Ni composites. We attribute this to a couple of reasons. First, the thermal conductivity of pristine Cu (385 W m^−1^ K^−1^) is larger than pristine Ni (90 W m^−1^ K^−1^), which would reduce the impact of the graphene on the Cu/G/OD composite (Supplementary Note 4). Also, the Cu/G composite shows a larger amount of defects in the graphene compared with the Ni/G composite, as evidenced by the magnitude of the D peaks in the Raman spectra (Supplementary Fig. 2). A comparison of our thermal conductivity measurements with the H–S model^38–40^ supports that a reduction in interfacial thermal resistance between the metal foam and the OD, as imparted by the graphene, is likely the main factor for the thermal conductivity enhancement of the composites (Supplementary Note 4).

### Application in thermal resonance devices

**Fig. 3:**
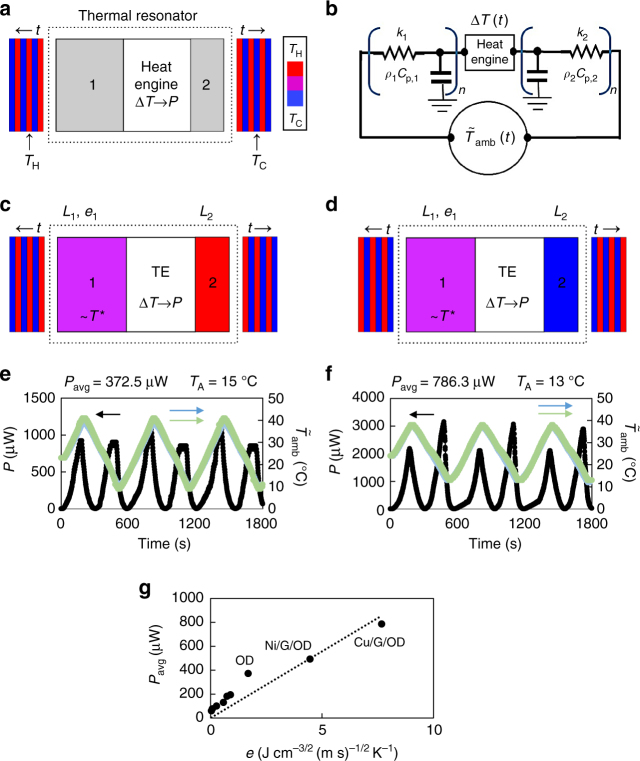
Application of ultra-high thermal effusivity materials in a thermal resonator. **a** A general schematic of a thermal resonator. A heat engine is encased between two thermal masses (1 and 2), which convert input temperature fluctuations—shown here as oscillating in time (*t*) between hot (red; *T*_H_ = *T*_0_ + *T*_A_) and cold (blue; *T*_C_ = *T*_0_ − *T*_A_)—into a spatial temperature difference, $\Delta T(t)$, that is converted to power (*P*) by the heat engine. **b** A thermal circuit demonstrating the operation and modeling details of a thermal resonator. Input, temperature fluctuations, $\tilde T_{{\mathrm{amb}}}(t)$, are applied and transformed into a spatial temperature difference by tuning the system’s thermal resistances $(k_1,\,k_2,\,L_1,\,L_2)$ and capacitances $(\rho _1C_{{\mathrm{p}},1},\,\rho _2C_{{\mathrm{p}},2})$. **c**, **d** General schematics of a thermal resonator resembling the devices constructed and tested in this work, which incorporate a high thermal effusivity PCM as thermal mass 1 and a negligible thermal mass as 2. The schematics are similar to **a**, except the heat engine has been replaced by a thermoelectric (TE) and the anticipated temperature distribution throughout the device has been shown. Thermal mass 2 quickly responds to the temperature of the environment (hot, **c** or cold, **d**). Thermal mass 1 has a phase transition temperature (T^*^) near the median temperature of oscillations and mainly exists at this temperature. **e** Power profile (black) and input temperature profiles (blue, green) for a thermal resonator incorporating OD as thermal mass 1 (Supplementary Note 7). The time-averaged power output (*P*_avg_) and temperature oscillation amplitude (*T*_A_) are also provided. **f** Power profile (black) and input temperature profiles (blue, green) for a thermal resonator incorporating Cu/G/OD as thermal mass 1 (Supplementary Note 7). The time-averaged power output (*P*_avg_) and temperature oscillation amplitude (*T*_A_) are also provided. **g** Time-averaged power output (*P*_avg_) of thermal resonators as a function of the thermal effusivity of thermal mass 1 (Methods, Supplementary Note 6). The linear fit of the data with the intercept forced through the origin is also shown, and the slope of the fit is 111, with the units determined by the axes. The six unlabeled data points in ascending order of thermal effusivity correspond to styrofoam, neoprene foam, wood, PVC, Teflon, and neoprene rubber. For PCMs (OD, Ni/G/OD, Cu/G/OD), thermal effusivity is given by Supplementary Eq. (16)

A general schematic of a thermal resonance device is shown in Fig. 3a. The generic device consists of a heat engine encased between two thermal masses, which convert temperature fluctuations to a spatial temperature difference for power extraction via the heat engine. Consider the thermal circuit depicted in Fig. 3b, where the ambient thermal environment is taken to be a time varying source term:

3 $$\tilde T_{{\mathrm{amb}}}(t) = T_0 + T_{\mathrm{A}}\sin \left( {\omega t} \right),$$

where $\tilde T_{{\mathrm{amb}}}$ is the ambient temperature, *T*_0_ is the median ambient temperature, *T*_A_ is the amplitude for ambient thermal fluctuations, *ω* is the angular frequency of temperature oscillations, and *t* is time.

This source is connected to a generic heat engine capable of the conversion of a temperature difference, $\Delta T(t)$, to usable work. As a specific example, consider a thermoelectric device, where a temperature difference approximately yields the power output:^41^

4 $$P = \frac{{\left( {\Gamma _{\mathrm{S}}\Delta T\left( t \right)} \right)^2}}{{R_{{\mathrm{eff}}}}},$$

where *P* is power, $\Gamma _{\mathrm{S}}$ is the Seebeck coefficient, and *R*_eff_ is an effective resistance that accounts for thermal and electrical resistances in the system^41^. Note that in the absence of additional thermal circuit elements, the power generated is zero for all time. However, as in the case of the circuit in Fig. 3b, the heat engine is encased between two thermal masses (1 and 2), having constant thermal conductivities ($k_1,\,k_2$) and thermal capacitances ($\rho _1C_{{\mathrm{p}},1},\,\rho _2C_{{\mathrm{p}},2}$). These resistive and capacitive elements are differential in size $(n \to \infty )$ and form a finite transmission line^42^. The linear thermal masses can be specifically paired to maximize the spatial and temporal temperature gradient existing across the heat engine, such that persistent power can be harvested according to Eq. (4) from temperature fluctuations.

To optimize the power harvesting capabilities of thermal resonance devices, we postulate for thermal mass 1 (dominant thermal mass) to be tuned to the temperature oscillation frequency, while thermal mass 2 should have negligible thermal resistance (extrinsic). We also postulate that maximizing the thermal effusivity (intrinsic) of thermal mass 1 will optimize the power output. As we show later, these design criteria fall into the optimal performance regime. Furthermore, because our thermal resonators operate in a dynamic temperature environment, we can select a PCM with a transition temperature in proximity to the median temperature of oscillations and evoke Eq. (2) for an enhanced thermal effusivity. Figure 3c, d illustrates the thermal resonator’s operation under such constraints. Thermal mass 2 rapidly responds to the temperature of the ambient environment and adopts its temperature, whereas the dominant thermal mass does not rapidly respond to the environment and exists mainly at the phase transition temperature of the high thermal effusivity PCM. Throughout this paper, we will optimize thermal mass 1, while maintaining an invariant thermal mass 2, which is characterized by low thermal resistance.

The beneficial effects of high thermal effusivity materials toward thermal energy harvesting via thermal resonators, in terms of power output, are demonstrated experimentally in Fig. 3e–g. The oscillatory temperature inputs, which mimic ambient temperature fluctuations, are generated using a programmable, temperature-controlled stage (Methods, Supplementary Fig. 9). The power output profiles for various resonators—incorporating thermal masses ranging from standard masses (e.g., Styrofoam, PVC, Teflon, etc.) to our high thermal effusivity materials—are measured, and the time-averaged power outputs (proportional to the extracted energy for a given time frame) are plotted with respect to the thermal effusivities of the dominant thermal masses (Fig. 3g, Supplementary Notes 5, 6, Supplementary Fig. 10). Typical power output profiles and input temperature oscillations for OD and Cu/G/OD thermal resonators are shown in Fig. 3e, f, respectively. Note the temperature oscillates around the phase transition temperature of our high effusivity materials, which enable activation of the enhanced, effective thermal effusivity definition in Eq. (2). As a control, we also investigate the performance of the device in the absence of thermal masses 1 and 2 (Supplementary Fig. 9), and the results show insignificant power generation compared to our tuned devices. Furthermore, the average power output of each device is closely related to the thermal effusivity (Fig. 3g, mathematical details in the following section). Comparisons of the average power vs. thermal conductivity and thermal capacity (Supplementary Fig. 11) do not show significant trends. The maximum average power that we observe is 786 μW for the Cu/G/OD resonator. This value can be compared with previous efforts to harvest from temperature fluctuations using non-PCMs^23–27^ and unmodified PCMs^30,31^ with thermoelectrics by considering the power output of the non-PCMs and pure OD in Fig. 3g. For these simulated temperature oscillations, we also calculated the anticipated average power output of similarly sized, common (250 μW) and state-of-the-art (490 μW) pyroelectrics for comparison to our thermal resonators (Supplementary Note 8).

### Theoretical design of a tuned thermal resonator

To model the thermal circuit shown in Fig. 3b, we start with the continuum energy conservation equation for a linear, 1-D system:

5 $$\frac{{\partial T_j}}{{\partial t}} = \alpha _j\frac{{\partial ^2T_j}}{{\partial x_j^2}},\quad \left( {j = 1\,{\mathrm{or}}\,2} \right),$$

where *T*_*j*_ is the spatial and temporal temperature of thermal mass *j*, *α*_*j*_ refers to the thermal diffusivity of thermal mass *j*, and *x*_*j*_ refers to the spatial dimension of thermal mass *j*.

Equation (5) completely describes the diffusion of heat for the non-PCMs in our thermal resonator. However, in modeling the PCMs in our devices, the following assumptions are made in order to apply Eq. (5): thermal fluctuations exist around the phase transition temperature and within the melting range of the PCM^43^ and thermal diffusion occurs due to the movement of the phase change boundary (Supplementary Note 1). The constant thermal diffusivity that we assume in this work appears to adequately describe the frequency response of our high effusivity materials.

We are interested in the open circuit characterization—an ideal scenario—of the device, and hence the limit of zero heat flux at the interfaces between the thermal masses and the heat engine. In addition, external convective heat transfer resistances and radiation are neglected in the definitions of the boundary conditions:

6 $$T_j\left( {x_j = 0} \right) = \tilde T_{{\mathrm{amb}}}(t) = T_0 + T_{\mathrm{A}}\sin \left( {\omega t} \right),$$

7 $$\left. {\frac{{\partial T_j}}{{\partial x_j}}} \right|_{x_j = L_j} = 0,$$

where *L*_*j*_ is the length of thermal mass *j*, *x*_*j*_ = 0 is the interface between thermal mass *j* and the ambient environment, and $x_j = L_j$ is the interface between thermal mass *j* and the heat engine.

Through separation of variables, we solve this linear system to yield the time and space distribution for the temperature profile of thermal mass *j*:

8 $$T_j\left( {x_j,t} \right) = T_0 + T_{\mathrm{A}}{\mathrm{Re}}\left[ {e^{i\omega t}} \right]\frac{{{\mathrm{Re}}\left[ {\cosh \left( {\sqrt {\frac{{i\omega }}{{\alpha _j}}} \left( {L_j - x_j} \right)} \right)} \right]}}{{{\mathrm{Re}}\left[ {\cosh \left( {\sqrt {\frac{{i\omega }}{{\alpha _j}}} L_j} \right)} \right]}}.$$

Equation (8) demonstrates that two different thermal masses will generate a temperature difference under fluctuating temperatures. A thermoelectric device situated between two such thermal masses will, therefore, generate electrical output according to Eq. (4).

**Fig. 4:**
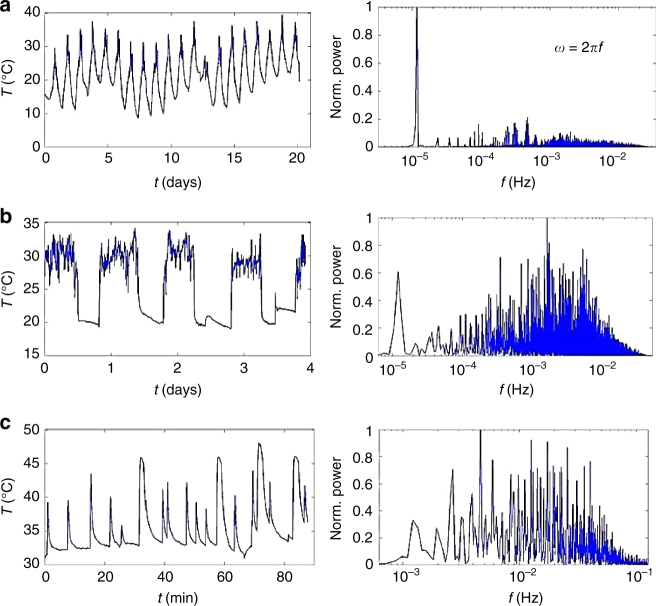
Examples of ambient and transient temperature baths. **a** Temperature profile (left) and FFT data (right) for a diurnal temperature cycle. **b** Temperature profile (left) and FFT data (right) for a wearable temperature sensor (SHTC1 Smart Gadget with Bluetooth^TM^ interface from Sensirion). **c** Temperature profile (left) and FFT data (right) obtained from the exhaust port of a Dell Latitude E6330 laptop

The design of a tuned thermal resonator is motivated by the frequency distribution of the specific ambient environment. We measure several examples of temperature fluctuations present in the environment (Fig. 4). The first measurements are of the diurnal temperature cycle as measured from an isolated backyard in Lexington, MA, USA, over a period of approximately 21 days in June 2014 (Fig. 4a, left). Temperatures range from approximately 10–38 °C, and a Fast Fourier Transform (FFT) of this data set yields the expected mode at 10^−5^ Hz (Fig. 4a, right). Diurnal temperature variation associated with pavement has also been identified as a harvestable energy source^44^. Figure 4b presents the temperature fluctuations experienced by an electronic device placed in the pocket of the principal investigator over a period of 4 days. The temperatures range from 20 to 32 °C during activity throughout the day. Note, the diurnal cycle is superimposed in the FFT (Fig. 4b, right). Others have also identified personal temperature fluctuations as a harvestable energy source^45^. Lastly, we measured the temperature fluctuations associated with the exhaust port of a laptop over a period of 90 min, resulting in a temperature profile related to the duty cycle of the heat exchanger (Fig. 4c, left). The breadth of the frequency distribution is a feature of such fluctuations that is treated in our models.

In order to effectively harvest energy from ambient temperature fluctuations, the theoretical model provided in Eq. (8) needs to be applied to the design of thermal resonators for given temperature oscillation frequencies, such as those provided in Fig. 4a–c. Qualitatively, this corresponds to tuning the thermal masses, in terms of thermal resistances and capacitances, to the temperature oscillation frequency to optimize power. A performance factor, *Q* can be derived from Eq. (8), which quantifies the time-averaged power output, *P*_avg_, of a thermal resonator design relative to the maximum power, *P*_max_, attainable given the amplitude of temperature fluctuations:

9 $$Q = \frac{{P_{{\mathrm{avg}}}}}{{P_{{\mathrm{max}}}}} = \frac{{\Delta T_{{\mathrm{avg}}}^2}}{{4T_{\mathrm{A}}^2}}= \frac{1}{8}\left( {{\mathrm{Re}}\left[ {{\mathrm{sech}}\left( {\sqrt {iv} } \right)} \right] - {\mathrm{Re}}\left[ {{\mathrm{sech}}\left( {\sqrt {Riv} } \right)} \right]} \right)^2,$$

where

10 $$v = \omega \frac{{L_1^2}}{{\alpha _1}},\,R = \left( {\frac{{\alpha _1}}{{\alpha _2}}} \right)\left( {\frac{{L_2^2}}{{L_1^2}}} \right).$$

**Fig. 5:**
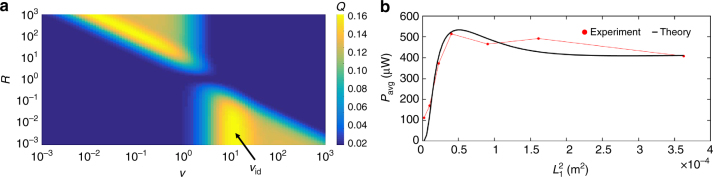
Theoretical and experimental design of a resonator for personal temperature fluctuations. **a** A heat map guiding the design and performance (*Q*) of a thermal resonance device, provided the dimensionless oscillation frequency $(v)$ of the ambient temperature bath and the ratio of thermal mass diffusion time scales (*R*). **b** Time-averaged power output (*P*_avg_) of thermal resonance devices as a function of the square of the length (*L*_1_) of the dominant thermal mass from experiment (red) and fitted with theory (black) (Methods, Supplementary Note 10). An input, angular temperature oscillation frequency ($\omega _{\mathrm{f}}$), which corresponds to the frequency of personal temperature oscillations (Fig. 4b), is applied to all systems and is indicated in Supplementary Fig. 13

A heat map of the performance factor as a function of the dimensionless temperature oscillation frequency, *v*, and the ratio of thermal diffusion time scales, *R*, for each thermal mass is shown in Fig. 5a. This heat map possesses symmetry in relation to *R*. Based on the choice of terming the high thermal effusivity material as thermal mass 1 and the low thermal resistance material as thermal mass 2, as well as our decision to non-dimensionalize the temperature oscillation frequency by thermal mass 1, the main region of interest in the heat map is the bottom portion $\left( {R \ll 1} \right).$ In this portion of the heat map it is apparent that there is an ideal *v*, *v*_id_, at which *Q* is optimized. We can solve for *v*_id_ and the maximum performance factor numerically after simplification of Eq. (9) in the limit *R *= 0:

11 $$Q = \frac{1}{8}\left( {{\mathrm{Re}}\left[ {{\mathrm{sech}}\left( {\sqrt {iv} } \right)} \right] - 1} \right)^2.$$

The maximum performance factor $(Q_{{\mathrm{max}}} = 0.16)$, which occurs at the ideal dimensionless temperature oscillation frequency $\left( {v_{{\mathrm{id}}} = 10.8} \right)$, indicates that approximately 16% of the maximum attainable power can be harvested with an optimized design. Furthermore, the ideal dimensionless temperature oscillation frequency indicates that, for a given temperature oscillation frequency, there is an ideal thermal diffusion time scale that should be tuned for thermal mass 1.

To verify the prediction of ideal frequency, we experimentally investigated the relationship between the thermal diffusion time scale of the dominant thermal mass and the ideal temperature oscillation frequency (Supplementary Fig. 12, Supplementary Methods). The setup shown in Supplementary Fig. 12 was used to probe the open circuit performance of three thermal resonator designs—with varying lengths of thermal mass 1—over a broad range of temperature oscillation frequencies. Based on the definition of *v* in Eq. (10), we expect the ideal dimensional frequency for operation to be inversely related to the time scale of thermal diffusion for thermal mass 1, which is indeed confirmed by the experimental data. Furthermore, the experimental data were fitted to the performance factor model in Eq. (9), as shown by the color-corresponding solid lines (Supplementary Note 9). Overall, the model developed for tuning the thermal resonator appears to agree with experimental data and can guide the design for harvesting energy from thermal fluctuations occurring in the ambient environment.

Equation (9) and the heat map in Fig. 5a provide insight to designing a power-optimized thermal resonator, in terms of length scales, for a given temperature fluctuation frequency and material for thermal mass 1. However, the criterion for selecting the material to act as thermal mass 1 to optimize power output has yet to be addressed mathematically. In order to investigate the power output of any general thermal resonator design, an estimate of the maximum attainable power, *P*_max_, is necessary. This parameter depends on the amplitude of the temperature fluctuations, and, most importantly, on the material of thermal mass 1^41^. The maximum attainable power density can be estimated by evaluating the maximum heat flux entering and exiting the thermal resonance device:

12 $$J_j = \left. { - k_j\frac{{\partial T_j}}{{\partial x_j}}} \right|_{x_j = 0},$$

where *J*_*j*_ is the thermal flux entering the external boundary of thermal mass *j*.

In the limit of *R*<<1, the maximum heat flux entering and exiting the thermal resonator is limited by the dominant thermal mass (*j *= 1) and is proportional to its thermal effusivity (Supplementary Note 10),

13 $$\left| {J_{{\mathrm{max}}}} \right| = e_1T_{\mathrm{A}}\sqrt \omega ,$$

where *e*_1_ is the thermal effusivity of the dominant thermal mass (*j *= 1).

Multiplication of the maximum heat flux entering and exiting the dominant thermal mass, $\left| {J_{{\mathrm{max}}}} \right|,$ with the performance factor and heat engine efficiency, *η*, yields an estimate of the average power density that is capable of being harvested, $p_{{\mathrm{avg}}},$ for a certain ambient environment and thermal resonator design (Eq. 14). In general, it is predicted that the average power output is proportional to the thermal effusivity of the dominant thermal mass for a constant value of *Q*. This occurs when $v \gg 1$ such that *Q* = 1/8, as in the case of the experiments in Fig. 3g, or it occurs when the thermal diffusion time scale (related to length and thermal diffusivity) is identical for the dominant thermal masses. It should also be noted that the average power density is closely related to the magnitudes of the efficiency of the thermoelectric, the amplitude of temperature fluctuations, and the frequency of temperature fluctuations.

14 $$p_{{\mathrm{avg}}} \approx \eta \left| {J_{{\mathrm{max}}}} \right|Q = \eta \,e_1T_{\mathrm{A}}\sqrt \omega \,Q.$$

Equation (14) is an estimate of the average power density output that is based on open circuit derivations. It is a simplified and general model that captures the trends of thermal resonator performance and guides the design of thermal resonators for particular environments. A rigorous derivation of the power output for a specific resonator design in a specific environment would require the selection of a particular thermoelectric and must be done numerically.

The data in Fig. 3g are linearly fitted to Eq. (14), and the slope, along with parameters related to the input fluctuations, can be used to estimate the efficiency of the thermoelectric (Supplementary Note 10). Note, tuning is held constant in Fig. 3g by constructing devices such that *v* is sufficiently greater than 1 (Supplementary Note 10). The estimated efficiency of the thermoelectric (0.3%) is within range of the maximum efficiency predicted by Supplementary Eq. (22) (1.7%). Overall, the estimate of the power density in Eq. (14) is well reflected by the data in Fig. 3g. The existence of a non-zero intercept in the experimental data is attributed to heat leakage via natural convection at the interface of the thermoelectric material and its ceramic plate (Supplementary Note 10).

With the model for the power density of a thermal resonator with respect to input temperature profiles, device tuning, and intrinsic device properties, we design and construct a thermal resonator using the Ni/G/OD composite that is tuned to the dominant frequency of personal temperature fluctuations (1 mHz) (Fig. 4b). The time-averaged power output for varying Ni/G/OD lengths, *L*_1_, is plotted and also fitted to Eq. (14) to evaluate the power generation model (Fig. 5b, Supplementary Note 10). An FFT of the simulated, input temperature boundary conditions for personal temperature fluctuations (Supplementary Fig. 13) is in agreement with Fig. 4b. The theoretical fit to the data predicts an effective thermal effusivity, 17.9 J cm^−3/2^ (m s K)^−^^1/2^, that is close to the value in Fig. 2 for the Ni/G/OD composite. Whereas the data in Fig. 3g hold tuning constant to probe the effect of effusivity and the data in Supplementary Fig. 12 neglect effusivity to probe tuning, the data in Fig. 5b combine the aspects of tuning and effusivity to completely probe the accuracy of the power generation model (Eq. 14). It should be noted that these results resemble the stored heat energy dependence of a PCM as the function of its thickness^46^. Overall, the model in Eq. (14) appears to describe the trend in the experimental data well, and suggests that both tuning and effusivity are paramount to optimal power generation via a thermal resonator.

## Discussion

**Fig. 6:**
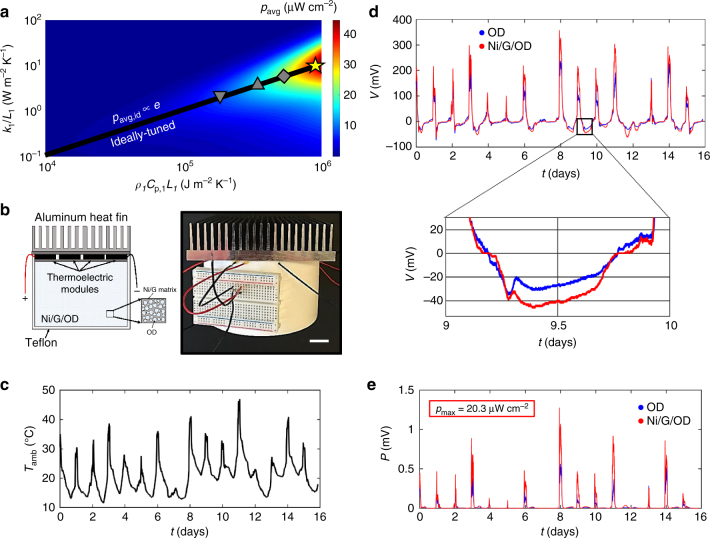
Reduction to practice of a power-optimized and tuned diurnal thermal resonator. **a** Heat map for the harvestable power density (*p*_avg_) from diurnal temperature fluctuations as a function of quantities related to the thermal resistance and thermal capacitance of thermal mass 1. The heat map assumes a diurnal temperature oscillation frequency $\omega = 7 \times 10^{ - 5}\,{\mathrm{s}}^{ - 1}$, $R \ll 1$, and a temperature oscillation magnitude *T*_A_ = 10 °C. The gray triangle $(\nabla )$, gray triangle (Δ), gray square, and yellow star correspond to ideally tuned dry soil, wet soil, OD, and Ni/G/OD thermal resonators, respectively. **b** Diurnal thermal resonance devices (schematic, left; photo, right) constructed from a hollow Teflon cylinder (height = 7.6 cm; diameter = 12.7 cm) and a 14 cm × 14 cm × 2.5 cm aluminum heat fin with four 4.0 cm × 4.0 cm Bi_2_Te_3_ commercial thermoelectrics wired in series as the heat engine situated between the thermal masses. The hollow Teflon cylinder contains stacked Ni/G/OD (26 pieces, diameter = 8 cm) as the phase change material. A control device containing pure OD was also constructed with the same dimensions (Methods, Supplementary Note 11). Scale bar: 2.5 cm. **c** Temperature profile of the transient, ambient environment where the diurnal thermal resonance devices were located over the course of 16 days in Cambridge, MA, USA, between 17 May and 2 June 2016. **d** Closed circuit voltage profiles for the diurnal thermal resonance devices when exposed to the ambient boundary conditions shown in **c**. **e** Power profiles for the diurnal thermal resonance devices when exposed to the ambient boundary conditions shown in **c**

For the reduction to practice of a thermal resonator in a non-simulated environment, we chose to target the diurnal cycle (Fig. 4a). Using Eq. (14) we generate a heat map for a diurnal environment that guides the practical design of the thermal resonator (Fig. 6a, Supplementary Note 11). Two variables, $k_1/L_1\,{\mathrm{and}}\,\rho _1C_{{\mathrm{p}},1}L_1$, related to the thermal resistance and thermal capacitance of thermal mass 1, for design manipulation in a given ambient environment, determine the value of $p_{{\mathrm{avg}}}$, and their product yields the square of thermal effusivity. The black line represents an ideally tuned thermal resonator—fulfilling the requirement that $v_{{\mathrm{id}}} = 10.8$—such that the time-averaged temperature difference across the heat engine is optimized in an open circuit limit. The harvestable power along this ideally tuned line is proportional to the thermal effusivity, as shown in Eq. (14). Our ultra-high thermal effusivity material (Ni/G/OD) introduced in this work, with dimensions tuned to that which optimizes the energy capture from the temperature oscillation frequency of the diurnal environment, is estimated to yield a power density of approximately 40 μW cm^−2^ (Fig. 6a, Supplementary Note 11). For comparison, the predicted power density outputs of ideally tuned dry soil, wet soil, and OD thermal resonators are also provided (Fig. 6a). The calculations for dry and wet soil thermal resonators are based on previous efforts to harvest from diurnal temperature fluctuations with a thermoelectric situated at a specific depth within the earth^24–27^. An upper threshold for the power density generated by the diurnal thermal resonator is estimated to be 330 μW cm^−2^ for a device limited by convection through the heat fin (Supplementary Note 12). Clearly, maximizing thermal effusivity is paramount to optimizing the harvestable power from temperature fluctuations using a thermal resonator and significantly enhances the power output in comparison to previous transient thermal energy harvesting approaches.

The thermal resonator theory was used to develop and construct thermal resonators capable of harvesting from the ambient temperature fluctuations of the diurnal cycle. A hollow Teflon cylinder—containing either OD or Ni/G/OD—was chosen as thermal mass 1, and an aluminum heat exchanger was chosen as thermal mass 2 (Fig. 6b, Supplementary Fig. 14, Methods). The devices’ power output and ambient temperature profile were monitored in outdoor conditions on the campus of MIT in Cambridge, MA, USA, over a period of 16 days (Methods). Figure 6c shows the diurnal temperature profile, and the devices’ voltage and power performances are shown in Fig. 6d, e, respectively. Temperature fluctuations centered around approximately 25 °C with amplitudes of approximately 10 °C yield voltage and power spikes of approximately 230 mV and 900 μW and 350 mV and 1.3 mW for the OD and Ni/G/OD devices, respectively. This corresponds to a maximum power density of 20 μW cm^−2^ and 2 μW cm^−3^ for the Ni/G/OD-based device. Furthermore, the devices generate power during the heating (daytime) and cooling (nighttime) cycles of the day, resulting in a persistent power source. The technology is persistent in the sense that the power (inset, Fig. 6d) is persistently non-zero with the exception of two points throughout the day—related to the times at which the cycles switch from heating to cooling and vice versa. As anticipated from theory, the device incorporating the ultra-high thermal effusivity material exhibited superior energy harvesting performance during heating and cooling (inset, Fig. 6d) cycles. As a quantitative comparison to theory in Eq. (14), it is anticipated that the enhancement in power output for the Ni/G/OD resonator compared to the OD resonator should be proportional to the thermal effusivity—a factor of 2.6 (Fig. 1c). The ratio of energy captured by the Ni/G/OD resonator compared to the OD resonator over the 16 day period (1.8), as calculated by the integration of both power profiles (Fig. 6e), is close to the anticipated ratio, and likely differs due to interfacial thermal resistances between the stacked Ni/G/OD samples (Supplementary Fig. 14). It should be noted that a ratio of the fitted effective thermal effusivity for Ni/G/OD (Fig. 5b) to that of OD (Fig. 1c) would yield 1.8, further suggesting that interfacial thermal resistances are likely lowering the power output. The fact that theory can inform this experimental design, which is enabled by materials science focused on maximizing the thermal effusivity, provides scientific and engineering approaches to improve the power densities that we report. This will be the focus of future efforts.

In this work, we develop ultra-high thermal effusivity materials for the dynamic capture and storage of ambient thermal energy. These materials are based on graphene-modified Cu and Ni foams, impregnated with a standard ambient PCM, OD. The incorporation of multi-layer graphene serves to significantly enhance the ability of the phase change composite to efficiently capture and store thermal energy in a dynamic manner. We identify and demonstrate that our ultra-high thermal effusivity materials are ideal candidates for a new form of ambient thermal energy harvesting via a device we call a broadband thermal resonator. Such devices, bolstered by high thermal effusivity materials, address the need for renewable energy sources that are not limited by intermittency, and capable of persistent operation. Ambient temperature fluctuations are analyzed for various, dominant frequencies, including the outdoor diurnal cycle at 10 μHz, fluctuations surrounding an active human body at 1 mHz, and a laptop computer duty cycle at 10 mHz. We develop a resonator theory that employs ultra-high thermal effusivity materials tuned to the frequency mode of environmental temperature oscillations to optimize the spatial and temporal gradient of thermal energy across a heat engine for persistent power extraction, and we show that the power harvested by these devices is proportional to the thermal effusivity of the dominant thermal mass. Lastly, we experimentally measure persistent energy harvesting from diurnal frequencies at an outdoor location for over 15 days. Such thermal resonance devices may provide energy sources over extended periods, particularly as high thermal effusivity materials and thermoelectrics are improved.

## Methods

### Fabrication of Ni/OD and Ni/G/OD

Two foams were impregnated with OD: pristine Ni foam substrate (porosity 95%, 80–110 PPI, thickness = 1.6 mm, MTI Corp.) and Ni foam (porosity 95%, 80–110 PPI, thickness = 1.6 mm, MTI Corp.) with CVD-grown multi-layer graphene—forming Ni/OD and Ni/G/OD, respectively.

Multi-layer graphene was grown upon the Ni foam substrate in a quartz tube of outer diameter 25 mm and inner diameter 22 mm. The Ni foams were exposed to Ar (300 sccm) for 15 min at ambient conditions. The Ni foams were then exposed to Ar (300 sccm) and H_2_ (100 sccm) for 20 min at ambient conditions. The Ni foams were then heated to 1000 °C and annealed for 30 min in a tube furnace (Lindberg Blue M) with Ar (300 sccm) and H_2_ (100 sccm) exposure. CH_4_ was introduced (10 sccm) for 5 min at 1000 °C as the carbon source for graphene growth. The sample was then quickly cooled to room temperature ~100 °C min^−1^ under Ar exposure (300 sccm). All steps occurred at 760 Torr.

The samples were vacuum impregnated when exposed to molten OD (Sigma-Aldrich) at approximately 40 °C in a vacuum oven for 2 h. Following impregnation, the samples were suspended and allowed to dry at ambient conditions.

Note, for constructing thermal resonators, passive impregnation was used to form Ni/G/OD.

### Fabrication of Cu/OD and Cu/G/OD

Two foams were impregnated with OD: pristine Cu foam substrate (porosity 95%, 580 μm nominal cell size, thickness = 1.9 mm, Alantum) and Cu foam (porosity 95%, 580 μm nominal cell size, thickness = 1.9 mm, Alantum) with CVD-grown multi-layer graphene—forming Cu/OD and Cu/G/OD, respectively.

Multi-layer graphene was grown upon the Cu foam substrate in a quartz tube of outer diameter 25 mm and inner diameter 22 mm. The Cu foams were exposed to Ar (300 sccm) for 20 min at ambient conditions. The Cu foams were then heated to 920 °C over the course of 30 min in a tube furnace (Lindberg Blue M) with Ar (300 sccm) exposure. CH_4_ (10 sccm) and H_2_ (30 sccm) were introduced for 90 min at 920 °C. The sample was then quickly cooled to room temperature ~100 °C min^−1^ under Ar (300 sccm) and H_2_ (30 sccm) exposure. All steps occurred at 760 Torr.

The samples were vacuum impregnated when exposed to molten OD (Sigma-Aldrich) at approximately 40 °C in a vacuum oven for 2 h. Following impregnation, the samples were suspended and allowed to dry at ambient conditions.

Note, for constructing thermal resonators, passive impregnation was used to form Cu/G/OD.

### Microscopic characterization

The structures of the materials were investigated with SEM (Zeiss Merlin high-resolution SEM; EHT = 10.00 kV; working distance = 13.3 mm; InLens signal; column mode=depth of field; current = 297 pA; 48× and 5760× magnifications) and Raman spectroscopy (Horiba Jobin Yvon Raman Microscope, excited at 532 nm).

### Thermal measurements

The thermal conductivity and latent heat of the materials were measured using the Transient Plane Source method (TPS 2500S from Hot Disk AB; at room temperature, *T *= 21 °C; Kapton 5501 sensor) and DSC (DSC Q100 from TA Instruments, −10 °C↔50 °C, 10 °C min^−1^), respectively. Refer to Supplemental Notes 2 and 3 for a more in-depth discussion of the Transient Plane Source method for measuring thermal conductivity.

The parameters and sample dimensions used for measuring the thermal conductivity of the samples are as follows:

OD—standard method; 175 mW; 10 s; analyzed points 56–159; samples (thickness ~2 cm, cross-section length ~5 cm and width ~5 cm) situated on either side of sensor; one sample with five measurements; measurements averaged and 95% confidence interval reported as the error.

Ni/OD and Ni/G/OD—thin slab method; 100 mW; 10 s; analyzed points 40–100; samples (thickness = 1.6 mm, diameter = 6 cm) situated on either side of sensor; four samples (Ni/OD) and four samples (Ni/G/OD) with three measurements for each sample; measurements averaged and 95% confidence interval reported as the error.

Cu/OD and Cu/G/OD—thin slab method; 100 mW; 5 s; analyzed points 75–150; samples (thickness = 1.9 mm, diameter = 6 cm) situated on either side of sensor; two samples (Cu/OD) and three samples (Cu/G/OD) with ten measurements for each sample; measurements averaged and 95% confidence interval reported as the error.

Thermal conductivity data for Ni/OD, Ni/G/OD, Cu/OD, and Cu/G/OD are provided in Supplementary Figs. 4–7.

For the reported values of latent heat, two scans were performed for each sample. The total samples for each type of material are as follows: OD (1), Ni/OD (1), Ni/G/OD (1), Cu/OD (2), and Cu/G/OD (3). The latent heat values for each type of material were averaged and a 95% confidence interval was reported as the error.

### Closed circuit-simulated thermal resonator experiments

Closed circuit thermal resonator experiments were performed with the setup shown in Supplementary Fig. 9 (Supplementary Note 6). A U-shaped Cu strip (thickness ~ 1 mm) is contacted with a temperature-controlled, programmable stage (Temperature Controlled Microscopic Stage from Linkam Scientific) using thermal paste to provide parallel oscillating temperature boundary conditions to a thermal resonance device. The temperature-controlled stage is programmed to oscillate between 0 and 50 °C at a scan rate of 10 °C min^−1^. Thermal contact resistances limit the upper and lower input temperatures that the thermal resonance device achieves, refer to Fig. 3. The temperature oscillation frequency of the simulated environment is shown in Supplementary Fig. 13 and corresponds to that of personal temperature fluctuations (Fig. 4b). The Cu strip is also in direct contact with a thermoelectric (Custom Thermoelectric; 03111-5L31-03CF; 1.5 cm × 1.5 cm) on one side using thermal paste, and on the opposing side, the Cu strip contacts thermal mass 1 using thermal paste. The output closed-circuit voltage of the thermal resonance device is monitored over a 1.5 Ω resistor (impedance matched to thermoelectric; Supplementary Note 7) using an oscilloscope (DrDAQ data acquisition board). Descriptions and schematics of the thermal masses used in Figs. 3 and 5 are provided in Supplementary Note 6 and Supplementary Fig. 10.

### Reduction to practice of thermal resonator

A hollow Teflon cylinder $(L_1 = 7.6\,{\mathrm{cm}},\,{\mathrm{diameter}} = 12.7\,{\mathrm{cm}})$—containing either OD or Ni/G/OD—was chosen as thermal mass 1, and an aluminum heat exchanger $(L_2 = 2.5\,{\mathrm{cm}},\,14\,{\mathrm{cm}}\,{\mathrm{x}}\,14\,{\mathrm{cm}})$ was chosen as thermal mass 2 (Fig. 6b, Supplementary Fig. 14). Four 4.0 cm × 4.0 cm commercial Bi_2_Te_3_ thermoelectrics (TEG2-126LDT) wired in series were chosen as the devices’ heat engines. The thermolectrics were wired to a 100 Ω resistor (approximately impedance matched, Supplementary Note 11), and the devices’ power output and ambient temperature profile were monitored using a DrDAQ data acquisition board in outdoor conditions on the campus of MIT in Cambridge, MA, USA, over a period of 16 days (17 May–2 June 2016). The devices were not directly exposed to the environment by covering in a black tarp. The devices were also not in proximity to thermal sources.

### Data availability

The data that support the findings of this study are available from the corresponding author upon reasonable request.

## Electronic supplementary material

Supplementary Information
