# Supplementary material for: Ultra-high thermal effusivity materials for resonant ambient thermal energy harvesting
Source: Nat Commun. 2018 Feb 14;9:664. doi: 10.1038/s41467-018-03029-x (PMC5813115; doi:10.1038/s41467-018-03029-x)
Supplement: Supplementary file 1 — Supplementary Information [file 41467_2018_3029_MOESM1_ESM.pdf]

Supplementary Information:

Supplementary Figures:

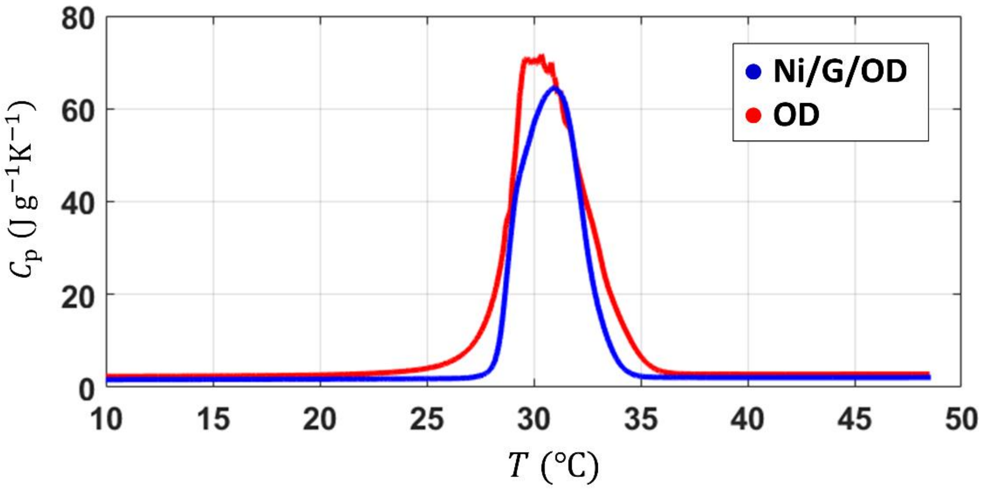

Supplementary Figure 1: Heat capacity associated with phase change. Specific heat data obtained for two samples (Ni/G/OD and OD) at a temperature scan rate of 5 ℃ min^{-1} using DSC. Experimental details provided in Supplementary Methods section below.

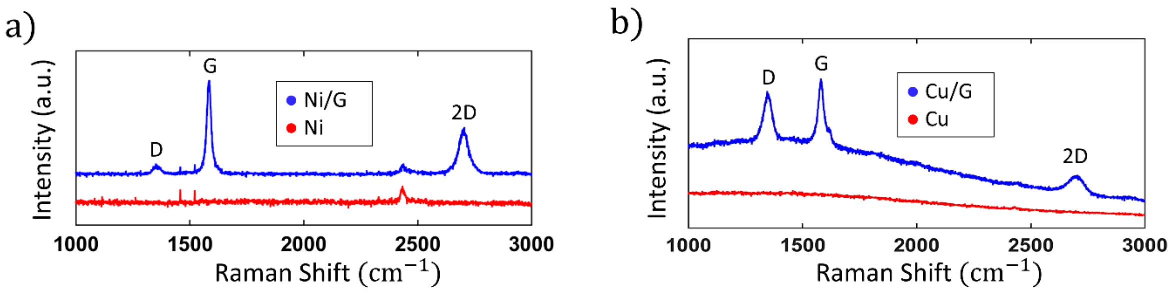

Supplementary Figure 2: Raman spectra for pure and graphene-modified metal foams. a) Raman spectra for pristine Ni foam and Ni foam with multi-layer graphene, Ni/G. b) Raman spectra for pristine Cu foam and Cu foam with multi-layer graphene, Cu/G.

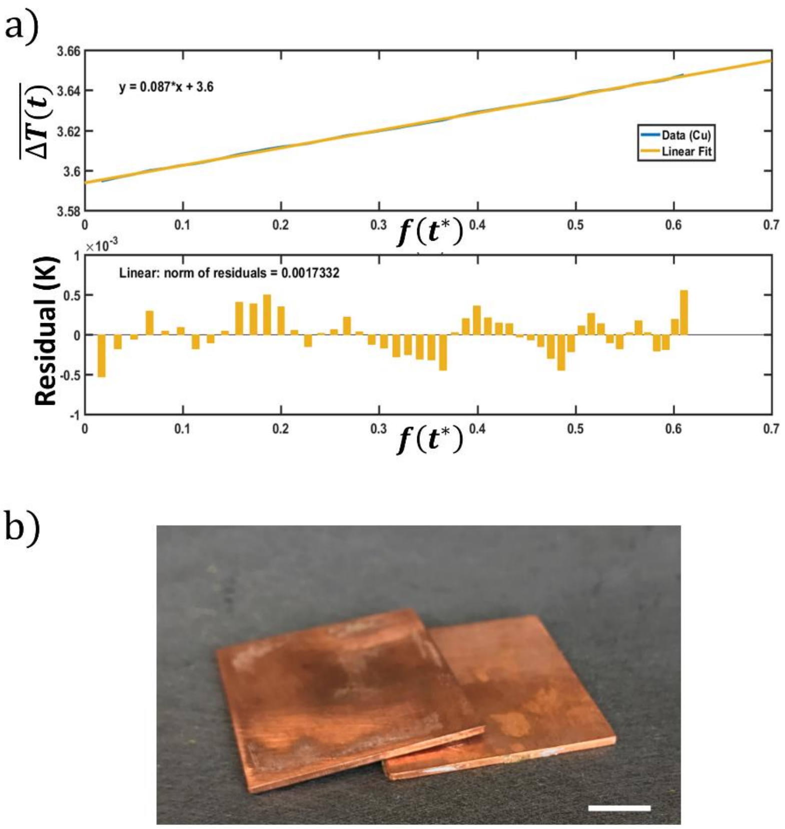

Supplementary Figure 3: Thermal conductivity of thin copper control samples. a) Thin slab results and fit residuals for thin (1.5 mm), square samples of solid copper (area 25 cm^2). The predicted thermal conductivity, as obtained from the fitted slope, is 385.7 W m^{-1} K^{-1}. Measurement settings: $P_0 = 1.2$ W, 1 second time interval for measurement, analyzed data points 50-100. b) Photo of the square, solid copper samples measured in a). Scale bar: 1.5 cm.

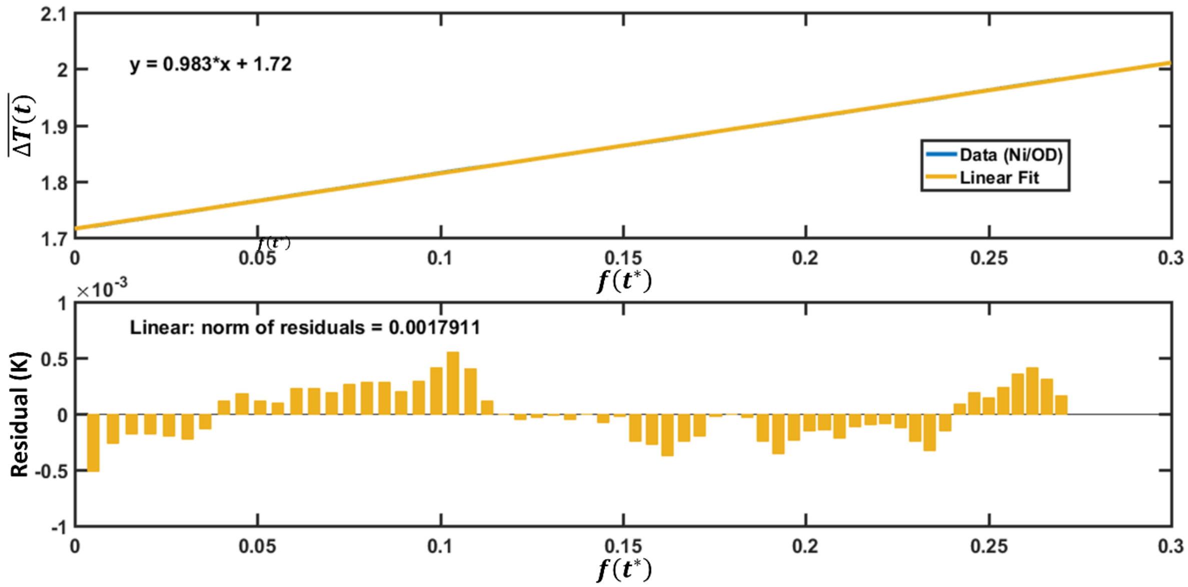

Supplementary Figure 4: Thermal conductivity of high effusivity materials. Ni/OD: $P_0 = 100$ mW, 10 second time interval for measurement, analyzed data points 40-100. Calculated k = 2.8525 W m^{-1} K^{-1}.

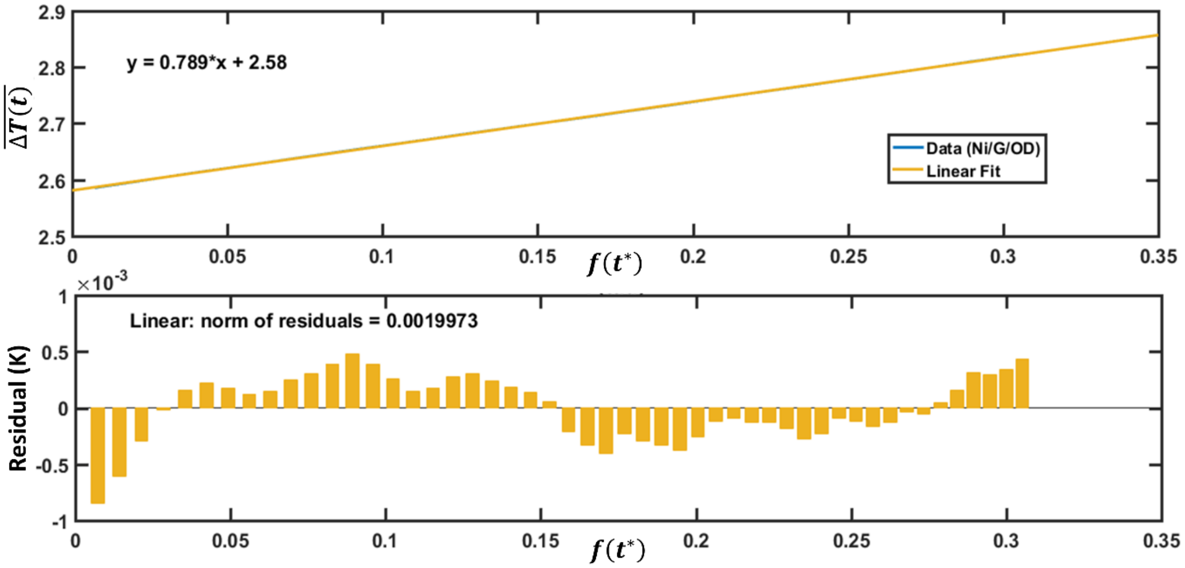

Supplementary Figure 5: Thermal conductivity of high effusivity materials. Ni/G/OD: $P_0 =$ 100 mW, 10 second time interval for measurement, analyzed data points 40-100. Calculated k = 3.5569 W m^{-1} K^{-1}.

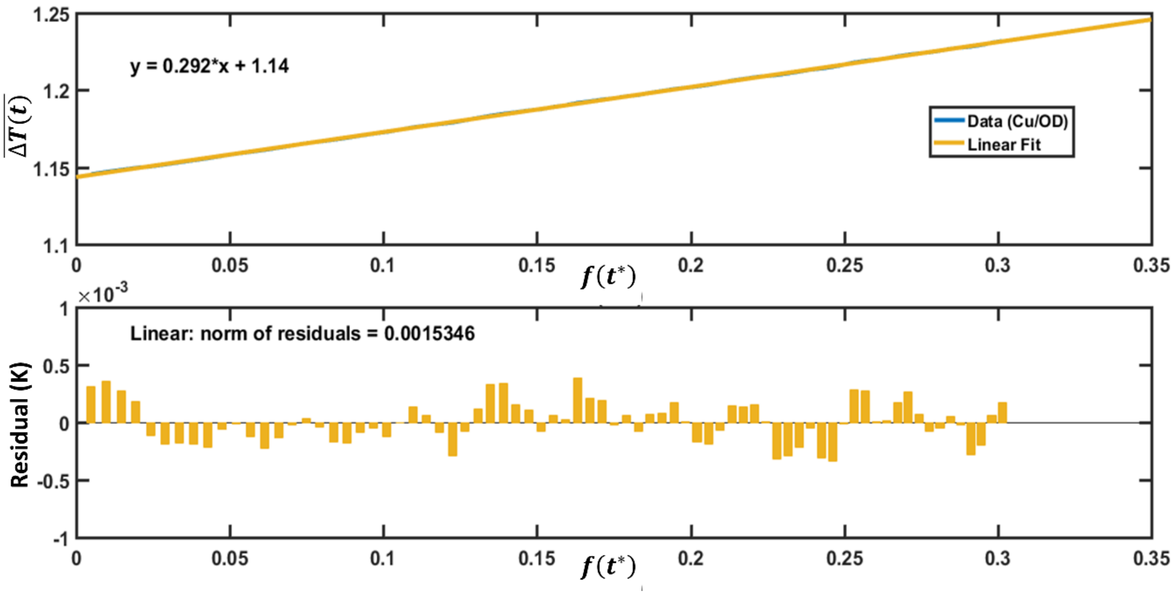

Supplementary Figure 6: Thermal conductivity of high effusivity materials. Cu/OD: $P_0 = 100$ mW, 5 second time interval for measurement, analyzed data points 75-150. Calculated k = 9.6111 W m^{-1} K^{-1}.

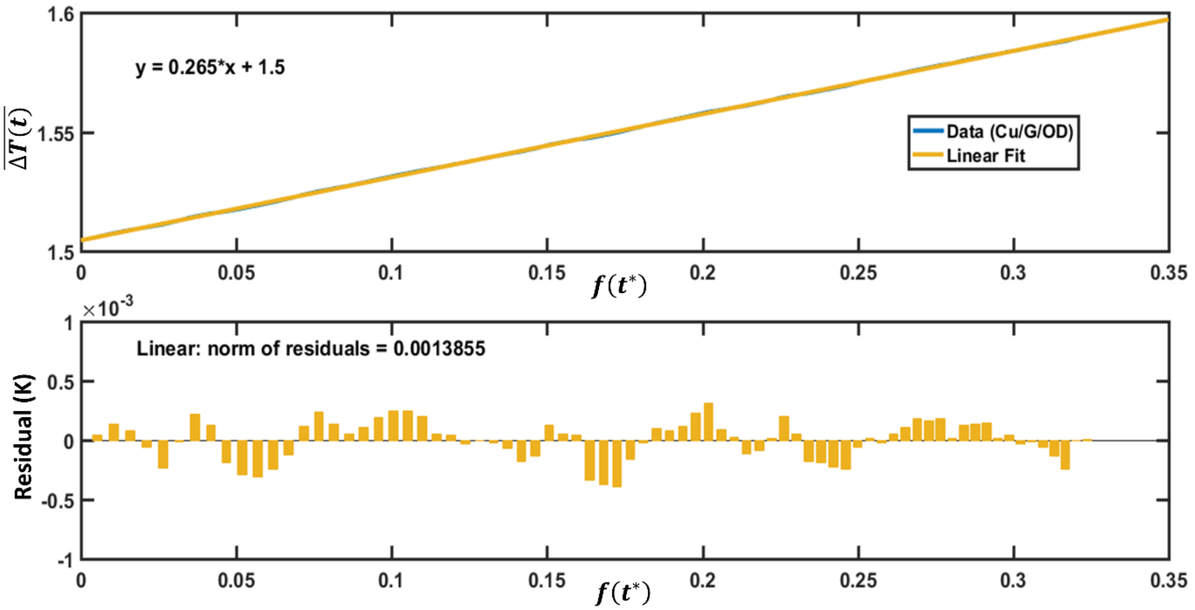

Supplementary Figure 7: Thermal conductivity of high effusivity materials. Cu/G/OD: P_0 = 100 mW, 5 second time interval for measurement, analyzed data points 75-150. Calculated k = 10.5992 W m^{-1} K^{-1}.

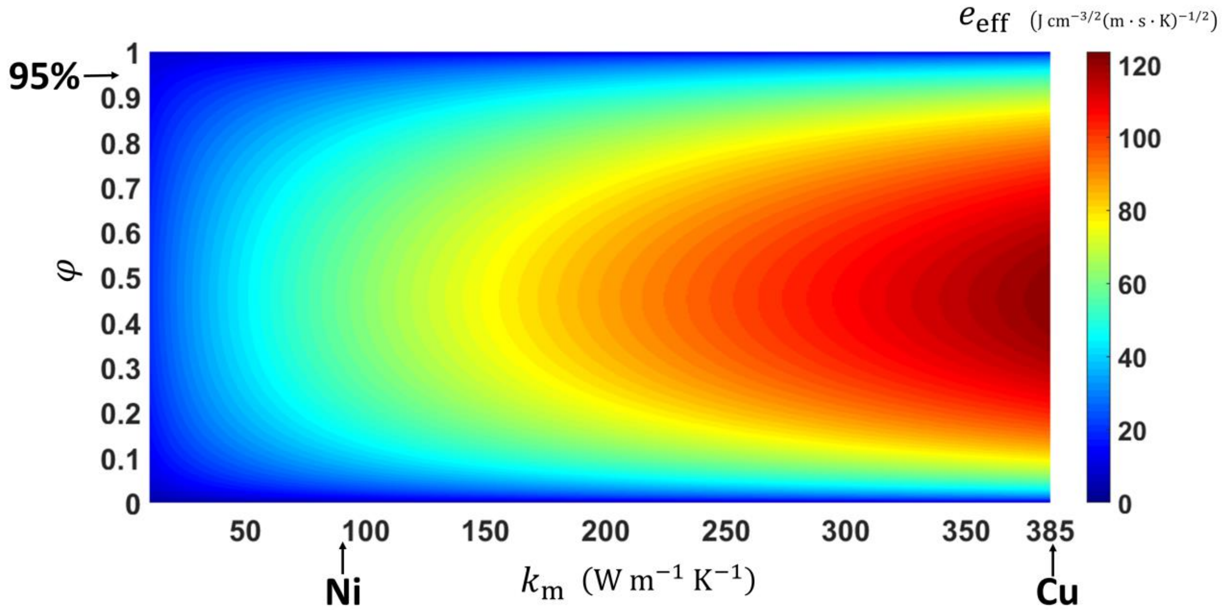

Supplementary Figure 8: Effusivity heat map based on theory. A heat map generated from Supplementary Eq. (11) for an OD-based metal foam composite. The effective thermal effusivity e_{eff} is plotted as a function of the metal foam's porosity φ and pure metal thermal conductivity k_{m}. The markers Ni, Cu, and 95% indicate the locations for our Ni and Cu composites.

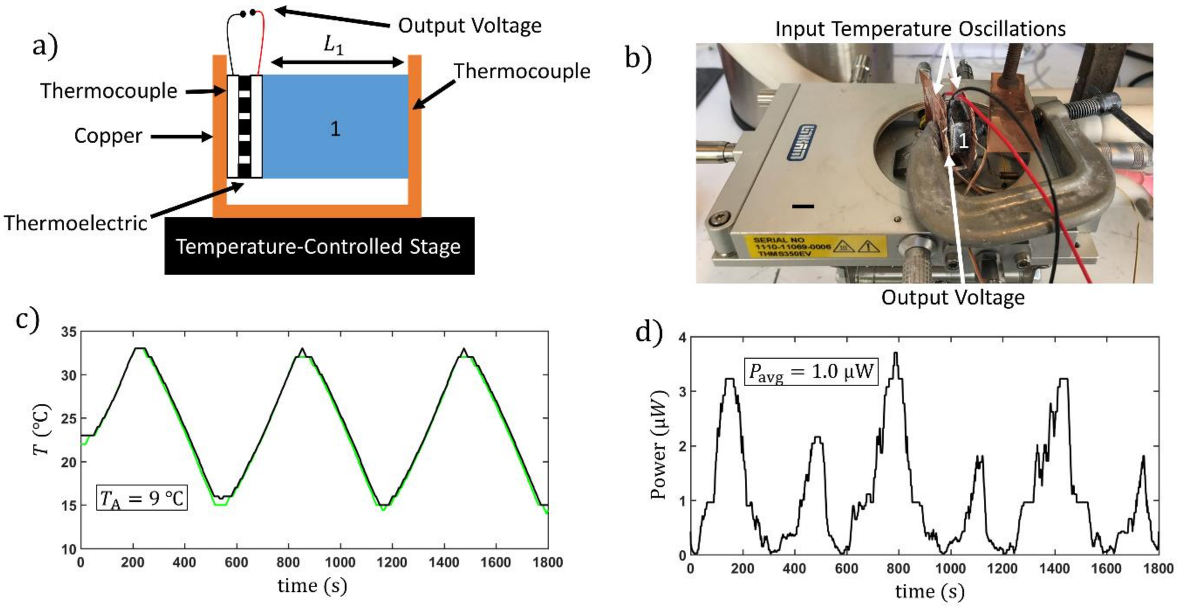

Supplementary Figure 9: Closed circuit simulated resonator experiments. a) Schematic of the setup used to simulate the oscillating temperature environment in Figs. 3 and 5. A U-shaped copper strip (thickness ~ 1 mm) is contacted with a temperature-controlled, programmable stage (Temperature Controlled Microscopic Stage from Linkam Scientific) to provide parallel oscillating temperature boundary conditions to a thermal resonance device. The copper strip is in direct contact with the thermoelectric on one side, and on the opposing side, the copper strip contacts thermal mass 1. The output closed-circuit voltage of the thermal resonance device is monitored over a 1.5 Ω resistor. The locations of K-type thermocouples encased in thin Kapton films are also shown, which are used to measure the input temperature oscillations to the thermal resonance device. b) Photo of the simulated, oscillating temperature environment setup for the thermal resonator that is depicted in a). Scale bar: 1 cm. c) Input temperature oscillations for a thermal resonator control, which corresponds to a bare thermoelectric in the absence of thermal masses 1 and 2. d) The power output of a thermal resonator control, which corresponds to a bare thermoelectric in the absence of thermal masses 1 and 2.

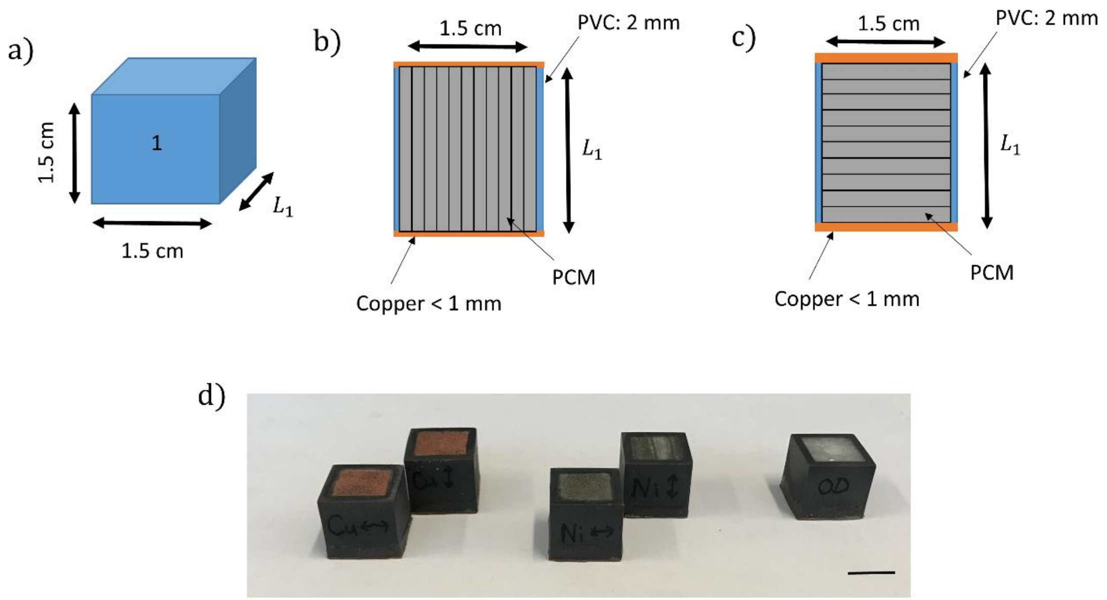

Supplementary Figure 10: Simulated resonator designs. a) Schematic of the thermal masses (1) employed to generate the data in Figs. 3 and 5. All masses had a cross-section (1.5 cm x 1.5 cm) with a varying length (L_1). b) Schematic of the thermal mass 1 used to generate the data in Figure 3. A hollow polyvinyl chloride (PVC) rectangular prism with thin copper tops was used. The phase change material – in the case of our composites – was stacked in parallel with the heat flow to more closely reflect the measured thermal effusivities. L_1 was constant for these devices at 1.3 cm. For non-phase change materials (e.g., Teflon, wood, etc.), the same dimensions were used for the pure material. c) Schematic of the thermal mass 1 used to generate the data in Fig. 5. A hollow PVC rectangular prism with thin copper tops was also used. The phase change material (Ni/G/OD) was stacked in series relative to the direction of heat flow. This was for ease of fabrication, as the devices lengths were varied significantly and reached quite small dimensions. d) Photos of the devices (without copper tops) shown in a) b) and c). Cu refers to the Cu/G/OD composite and Ni refers to the Ni/G/OD composite. ↕ refers to a composite arranged as in b). ↔ refers to a composite arranged as in c). Scale bar: 1 cm.

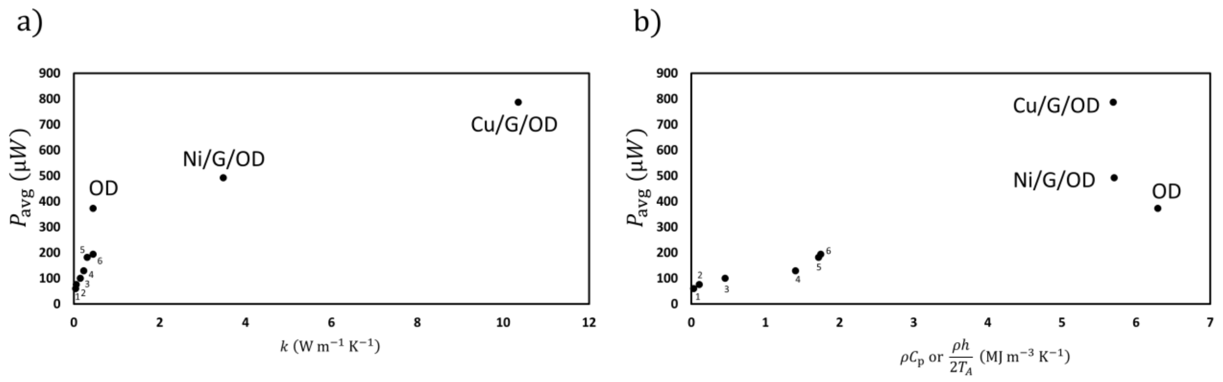

Supplementary Figure 11: Average resonator power output across designs. a) The time-averaged power output of the thermal resonance devices in Fig. 3g as a function of the material's thermal conductivity. Legend: (1-styrofoam, 2-neoprene foam, 3-wood, 4-PVC, 5-Teflon, 6-neoprene rubber). b) The time-averaged power output of the thermal resonance devices in Fig. 3g as a function of the material's thermal capacity. Legend: (1-styrofoam, 2-neoprene foam, 3-wood, 4-PVC, 5-Teflon, 6-neoprene rubber) (Supplementary Methods).

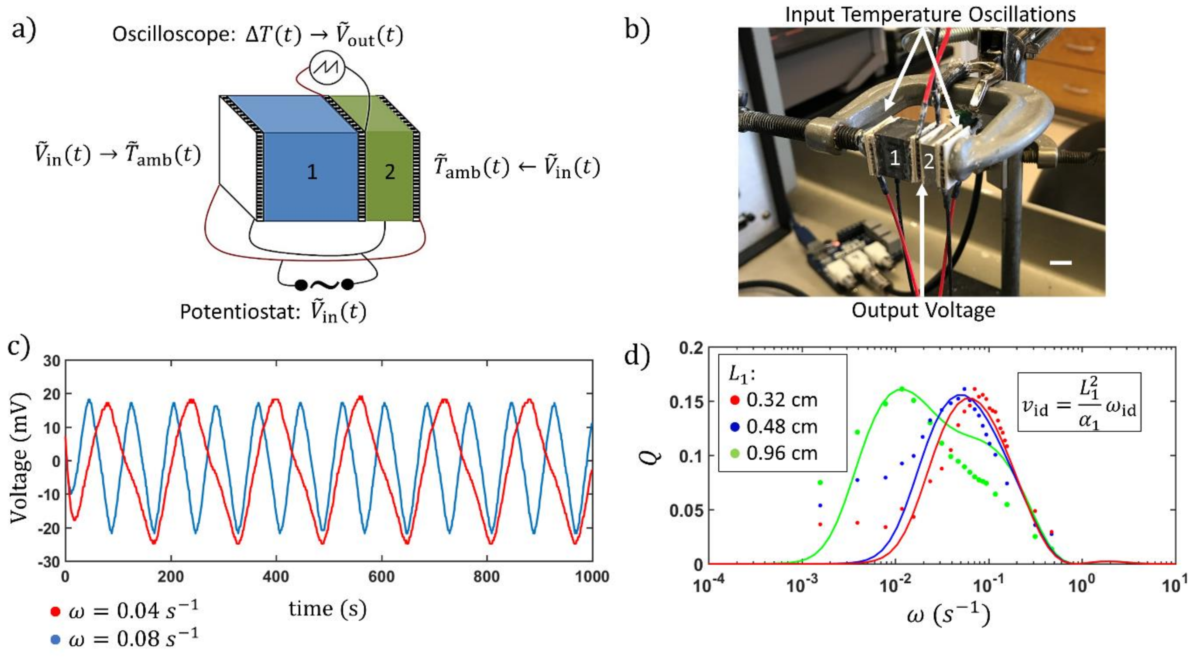

Supplementary Figure 12: **Open circuit simulated resonator experiments**. a) Schematic of the experimental setup for measuring the open circuit voltage output and performance of a thermal resonator. Two 1.5 cm x 1.5 cm Bi_2Te_3 commercial thermoelectrics are connected in parallel to a potentiostat and supply identical, oscillating temperature boundary conditions to either side of the thermal resonator. An additional 1.5 cm x 1.5 cm Bi_2Te_3 thermoelectric, acting as a heat engine, is inserted between specifically-paired thermal masses 1 and 2. An oscilloscope is used to measure the voltage output of the thermoelectric that acts as a heat engine. b) Photo of the experimental setup described in a). Scale bar: 1 cm. c) Open circuit voltage data with respect to time generated with the experimental setup in a) using a Teflon thermal mass of thickness 0.48 cm at two different temperature oscillation frequencies. d) Experimental (●) and analytical (−) results for the open circuit thermal resonators with Teflon at various thicknesses as thermal mass 1 (Supplementary Methods). Experimental data are fit to theory.

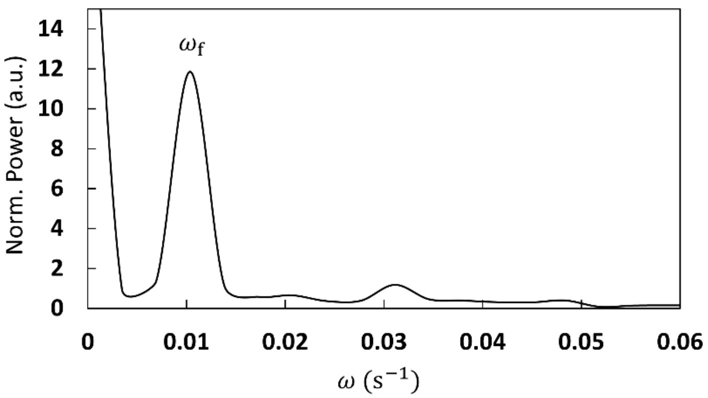

Supplementary Figure 13: Input temperature oscillation frequency for simulated experiments. The input, driving temperature fluctuation frequency (ω_f) for the closed circuit resonator experiments is shown in the FFT of the input temperature fluctuation data above.

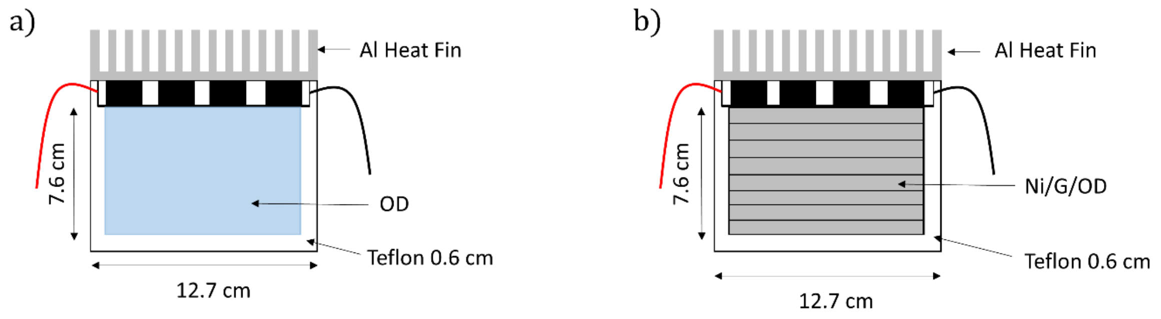

Supplementary Figure 14: Diurnal resonator reduced to practice. a) A schematic of the OD diurnal thermal resonator employed in Fig. 6. b) A schematic of the Ni/G/OD diurnal thermal resonator employed in Fig. 6.

Supplementary Tables:

Supplementary Table 1: Raw data for effusivity master plot. Data used to construct the master plot of effective thermal effusivity for phase change materials measured in the literature and this work, which is shown in Fig. 2. 'References' correspond to reference numbers in the main text and Fig. 2. 'SI References' refer to the Supplementary References. Results are confined to isotropic and ambient phase change materials (transition temperature T^* occurring between 20 and 40 ℃), for which volume-specific latent heat and thermal conductivity are reported. The four data points enclosed in the ellipse refer to this work for graphene-modified and pristine Ni and Cu foams impregnated with OD. The units for effective thermal effusivity (e_{eff}) match with the units in Fig. 2. The abbreviation EG represents expanded graphite.

Reference, SI Reference (Material)	k (W m^{-1} K^{-1})	ρ (g cm^{-3})	h (J g^{-1})	ρh (J cm^{-3})	T^* (℃)	e_{eff}
This Work (Ni/OD)	2.86	0.954	170	N/A	27.7	21.5
This Work (Ni/G/OD)	3.48	0.951	180	N/A	27.7	24.4
This Work (Cu/OD)	9.58	1.3	133	N/A	27.7	41.3
This Work (Cu/G/OD)	10.35	1.3	131	N/A	27.7	42.0
35, 1 (Capric-Palmitic-Stearic/EG)	5.225	0.743	128	N/A	20	22.3
34, 2 ($Zn(NO_3)_2$*$6H_2O$)	0.464	1.828	147	N/A	36	11.2
34, 2 (Polyglycol E600)	0.187	1.232	127	N/A	22	5.4
34, 2 (Paraffin C13-C24)	0.21	0.9	189	N/A	23	6.0
34, 2 (Paraffin C18)	0.148	0.774	244	N/A	28	5.3
34, 2 (Mistiric-Capric)	0.164	1.018	148	N/A	24	5.0
6, 3 (RT25-RT30)	0.19	0.785	232	N/A	26.6	5.9
6, 3 (OD)	0.19	0.865	244	N/A	27.7	6.3
6, 3 ($CaCl_2$*H_2O)	1.09	1.71	187	N/A	29.9	18.7
6, 3 (Na_2SO_4*$10H_2O$)	0.3	1.46	180	N/A	32	8.9
6, 3 (Paraffin Wax)	0.514	0.83	251	N/A	32	10.3
6, 3 (Capric Acid)	0.153	1.004	153	N/A	32	4.8
6, 3 (PEG900)	0.188	1.2	151	N/A	34	5.8
36, 4 ($CaCl_2$*$6H_2O$ $SrCl_2$ EG)	1.8	N/A	N/A	246	29	21.0
37, 5 (lauric-myristic-stearic EG)	2.51	0.55	134	N/A	29	13.6

Supplementary Notes:

Supplementary Note 1:

Apparent heat capacity associated with latent heat (Stefan formulation).

Consider a solid block that is initially ($t = 0$) entirely in the solid phase with a temperature equal to the phase change temperature, T^*, at which the solid transitions to a liquid state. For $t > 0$, the external boundary of the solid block is subjected to a constant temperature, T_b, where $T_\mathrm{b} > T^*$. The thickness (δ) of the moving solid-liquid phase change front is found to be:[6-7]

$$\delta(t) = 2At^{1/2}. \quad (1)$$

In the case of a heat transfer problem in which latent heat dominates sensible heat, the constant, A, is given by

$$A = \left(\frac{k_\mathrm{L}(T_\mathrm{b} - T^*)}{2\rho h}\right)^{1/2}, \quad (2)$$

where k_L refers to the thermal conductivity of the liquid phase, ρ refers to the density of the solid phase, and h is the latent heat per mass.

We can derive an equation for the apparent thermal diffusivity α_app associated with the moving phase change front:

$$\alpha_\mathrm{app} = \left(\frac{k}{\rho C_\mathrm{p}}\right)_\mathrm{app} = \frac{\delta^2}{t} = 4A^2 = \frac{2k_\mathrm{L}(T_\mathrm{b} - T^*)}{\rho h}, \quad (3)$$

where C_p is the heat capacity. An energy conservation model based on a temperature dependent heat capacity, which accounts for latent and sensible heat, was developed by Richardson et al. and solved numerically for a phase change material operating in a sinusoidally varying temperature bath around its phase transition temperature.[8] In the case of a Stefan number sufficiently greater than one – latent heat dominates sensible heat – their model reduces to Eq. (5) with a thermal diffusivity related to Supplementary Eq. (3). We estimate that our systems' values for the Stefan number are on the order of 10 or greater, depending on the magnitude of temperature fluctuations (≤ 10 °C) occurring around the phase transition temperature of our octadecane (OD) composites.[8,9]

In general, this comparison between standard heat capacity and latent heat by the incorporation of a temperature difference is the basis for the Stefan number, which is a common dimensionless number used in phase change heat transfer literature.[6]

The above equations illustrate the apparent heat capacity associated with latent heat for a phase change material in a dynamic environment, which operates in the vicinity of its phase transition temperature. The manifestation of this apparent heat capacity due to dynamic phase change is shown in Supplementary Fig. 1 for pure OD. A large amplification in the standard, thermodynamic heat capacity occurs due to the dynamic nature of the phase change. Furthermore, these equations demonstrate the applicability of a material's effective thermal effusivity towards evaluating its heat exchange with the surroundings when operating in a dynamic manner around its phase change temperature.

Supplementary Note 2:

Thermal conductivity measurements of standard materials and octadecane.

The Transient Plane Source (TPS) is a transient measurement technique for determining the thermal conductivity, thermal diffusivity, and heat capacity of a variety of types of materials.[10] The standard TPS technique has been widely employed for the measurement of phase change materials, as well as detecting thermal conductivity enhancements after nanomaterial and metal foam additions.[11-13]

The standard TPS measurement technique employs a plane source heating element, which also acts as a proxy for temperature measurement. The method assumes that the plane source is immersed in an infinite medium, and the relevant equations are shown below.

$$\overline{\Delta T(t)} = \frac{P_0}{\pi^{\frac{3}{2}} a k} f(t^*), \quad (4)$$

$$f(t^*) = \frac{1}{n^2(n+1)^2} \int_0^{t^*} \frac{1}{s^2} \sum_{l=1}^{n} l \sum_{j=1}^{n} j \exp\left(-\frac{\left(\frac{j}{n}\right)^2 + \left(\frac{l}{n}\right)^2}{4s^2}\right) I_0\left(\frac{\left(\frac{j}{n}\right)\left(\frac{l}{n}\right)}{2s^2}\right) ds, \quad (5)$$

where $\overline{\Delta T(t)}$ refers to the temperature increase of the sensor, P_0 refers to the power input of the sensor, a refers to the radius of the sensor, k refers to the thermal conductivity of the sample, t refers to time, t^* refers to a dimensionless time, n refers to the number of concentric rings in the sensor, and I_0 refers to the modified Bessel function.

As can be seen from Supplementary Eq. (4), a plot of $\overline{\Delta T(t)}$ vs. $f(t^*)$ should result in a linear relation with the slope being related to the thermal conductivity of the sample.

This standard method was used to measure the thermal properties of the standard materials in Fig. 3g and the pure OD sample.

We investigated the accuracy of the transient plane source technique for measuring the thermal conductivity of our samples by measuring the thermal conductivity of a NIST standard reference material: expanded polystyrene board (SRM 1453).[14] The certified thermal conductivity of the unit is given by Supplementary Eq. (6).

$$k = 0.00111 - 0.0000424\rho + 0.000115T, \quad (6)$$

where k is the thermal conductivity (W m^{-1} K^{-1}), ρ is the density (kg m^{-3}), and T is the temperature (K), which is valid from 281 K to 313 K.

We measured an average thermal conductivity of 0.03551 W m^{-1} K^{-1} with a standard deviation of 0.00009 W m^{-1} K^{-1} from five measurements at a temperature of 294 K with our transient plane source device. We used the settings P_0 = 15 mW for 80 seconds. The average density of the expanded polystyrene board is 41.5 kg m^{-3}, and from Supplementary Eq. (6), the expected thermal conductivity is 0.03316 W m^{-1} K^{-1}. Thus, we calculate an error of approximately 7% for our measurements.

Supplementary Note 3:

Thermal conductivity measurements of ultra-high thermal effusivity materials.

For the measurement of our ultra-high thermal effusivity materials, a modification of the standard TPS theory was employed.[15] This modification allows for the measurement of thin, highly thermally conductive materials, for which the assumption of an infinite medium in all dimensions collapses. For this method, termed the thin slab method, the highly thermally conductive, thin samples must be surrounded by sufficient insulation, in order to direct heat transport in a single plane throughout the thin samples. The mathematical theory for this method is summarized in Supplementary Eqs. (7) and (8).

$$\overline{\Delta T(t)} = \frac{P_0}{\pi^{\frac{3}{2}} a k} f(t^*), \tag{7}$$

$$f(t^*) = \frac{1}{n^2(n+1)^2} \int_0^{t^*} \frac{1}{s^2} \left\{ 1 + 2 \sum_{i=1}^{\infty} \exp\left[-\frac{i^2}{s^2} \left(\frac{\bar{h}}{a} \right)^2 \right] \right\} \sum_{l=1}^{n} l \sum_{j=1}^{n} j \exp\left(-\frac{\left(\frac{j}{n}\right)^2 + \left(\frac{l}{n}\right)^2}{4s^2} \right) I_0 \left(\frac{\left(\frac{j}{n}\right)\left(\frac{l}{n}\right)}{2s^2} \right) ds, \tag{8}$$

where $\bar{h}$ refers to the thickness of the thin sample on either side of the sensor.

As can be seen from Supplementary Eq. (7), a plot of $\overline{\Delta T(t)}$ vs. $f(t^*)$ should result in a linear relation with the slope being related to the thermal conductivity of the sample.

We verified the technique on our apparatus using two thin (1.5 mm), square samples of solid copper (area 25 cm^2) on either side of our sensor. The results and TPS settings are provided in Supplementary Fig. 3a, and a photo of the samples is shown in Supplementary Fig. 3b. As can be seen, a highly linear correlation was obtained, and the predicted thermal conductivity from this measurement was 385.7 W m^{-1} K^{-1}, which matches very well with literature.[16]

As indicated, the thin slab method was applied to our ultra-high thermal effusivity materials, and the results for Ni/OD, Ni/G/OD, Cu/OD, and Cu/G/OD are provided in Supplementary Figs. 4-7.

Supplementary Note 4:

Mechanism for thermal conductivity enhancement of ultra-high thermal effusivity materials.

We hypothesize that the thermal conductivity enhancement imparted by the incorporation of conformal graphene on the Ni and Cu foams results from two factors: reduced thermal interfacial resistance at the metal foam – OD interface and the ability of the graphene to bridge thermal transport across the grain boundaries of the metal foams. We believe that the former mechanism is the main driving force, due to the interface density of our foam and supporting literature.[17]

The thermal conductivities of our composites as measured via TPS are as follows – (Ni/OD, 2.9 W m^{-1} K^{-1}); (Ni/G/OD, 3.5 W m^{-1} K^{-1}); (Cu/OD, 9.6 W m^{-1} K^{-1}); (Cu/G/OD, 10.4 W m^{-1} K^{-1}). If we consider the upper bound as dictated by the Hashin-Shtrikman (HS) model,[18] which yields the highest possible thermal conductivity for an isotropic, two-phase composite, we obtain the following: (95% porous Ni foam with OD, 3.5 W m^{-1} K^{-1})[9,16,18] and (95% porous Cu foam with OD 13.4 W m^{-1} K^{-1}).[9,16,18] These upper bounds necessarily neglect the presence of interfacial resistance between the two phases of the composite. Considering our experimental data for the Ni and Cu composites with OD, it is apparent that we have not exceeded the HS upper bound, even for our graphene-incorporated composites. This fact supports the hypothesis that the interfacial resistance is a major factor in the thermal conductivity enhancement. In other words, if the CVD graphene had significantly altered the effective thermal conductivity of the ligaments of the metal foam (not an interfacial effect), we would anticipate to measure an effective thermal conductivity of the entire PCM composite that is in excess of the HS predictions provided above. Furthermore, recent work has supported this hypothesis.[17] Huang et al. also discovered a thermal conductivity improvement via interfacial thermal resistance reduction in nickel foam/epoxy composites with graphene oxide interfacial modifiers.[17]

Using the HS upper bound for thermal conductivity[18] and literature values for the thermal conductivity and latent heat of OD,[9] we can investigate the effect of the porosity of the metal foam on the effective thermal effusivity. Supplementary Eq. (9) provides the HS upper bound on thermal conductivity:

$$k_{\mathrm{HS,Upper}} = k_{\mathrm{m}}\left[\frac{2k_{\mathrm{m}}+k_{\mathrm{OD}}-2(k_{\mathrm{m}}-k_{\mathrm{OD}})\varphi}{2k_{\mathrm{m}}+k_{\mathrm{OD}}+(k_{\mathrm{m}}-k_{\mathrm{OD}})\varphi}\right], \quad (9)$$

where $k_{\mathrm{HS,Upper}}$ is the HS upper bound on thermal conductivity, k_{m} is the thermal conductivity of the pure metal composing the metal foam at room temperature, k_{OD} is the thermal conductivity of pure OD at room temperature $(0.36\ \mathrm{W\ m^{-1}\ K^{-1}})$,[9] and φ is the porosity of the metal foam.

Supplementary Eq. (10) provides the volumetric latent heat of a metal foam/OD composite as the porosity is varied:

$$(h\rho)_{\mathrm{eff}} = (h\rho)_{\mathrm{OD}}\varphi = (189\ \mathrm{J\ cm^{-3}})\varphi, \quad (10)$$

where $(h\rho)_{\mathrm{eff}}$ is the volumetric latent heat of the composite and $(h\rho)_{\mathrm{OD}}$ is the volumetric latent heat of the pure OD.[9]

Combining Supplementary Eqs. (9) and (10) yields the effective thermal effusivity (e_{eff}) of the composite:

$$e_{\text{eff}} = \left[(h\rho)_{\text{OD}}\varphi k_{\text{m}}\left[\frac{2k_{\text{m}}+k_{\text{OD}}-2(k_{\text{m}}-k_{\text{OD}})\varphi}{2k_{\text{m}}+k_{\text{OD}}+(k_{\text{m}}-k_{\text{OD}})\varphi}\right]\right]^{1/2}. \tag{11}$$

Using Supplementary Eq. (11), a heat map of effective thermal effusivity was generated for an OD-based metal foam composite as the metal matrix and porosity were varied (Supplementary Fig. 8). It is apparent that the effective thermal effusivity is maximized at a porosity of approximately 50%. In addition, maximization of the thermal conductivity of the pure component metal in the foam results in thermal effusivity enhancement. From the markers Ni, Cu, and 95%, which are present along the axes of the figure, it can be seen that the predictions from Supplementary Eq. (11) are in strong agreement with our experimental results. However, it should also be noted that our systems could have been further enhanced by tuning of the porosity of the metal foam.

Supplementary Note 5:

Thermal resonator design – relating heat capacity and latent heat.

For thermal resonator design, we desire to incorporate a phase change material which is characterized by a phase transition temperature that is equal to, or close, to the mean temperature of the temperature fluctuations - hence the applicability of our octadecane (OD) ($T^* = 27$ °C)[9] phase change composites towards thermal energy harvesting from ambient temperature fluctuations. The apparent thermal diffusivity associated with such a material operating in a fluctuating temperature environment can then be calculated from Supplementary Eq. (3) with the following modification:

$$(T_\mathrm{b} - T^*) = T_\mathrm{A}, \tag{12}$$

where T_A is the amplitude for temperature fluctuations.[8]

We then obtain the following equations for the apparent thermal diffusivity, apparent heat capacity, and apparent thermal effusivity:

$$\alpha_\mathrm{app} = \frac{2k_\mathrm{L}T_A}{\rho h}, \tag{13}$$

$$\left(\rho C_\mathrm{p}\right)_\mathrm{app} = \frac{\rho h}{2T_\mathrm{A}}, \tag{14}$$

$$e_\mathrm{app} = \sqrt{\frac{k_\mathrm{L}\rho h}{2T_\mathrm{A}}}. \tag{15}$$

The effective thermal effusivity in Eq. (2) is an intrinsic property of the material, and the apparent thermal effusivity – having identical dimensions to standard thermal effusivity – depends on the environment in which the material is applied. Hence, the relationship between the apparent thermal effusivity and effective effusivity – as shown below – must be applied in order to directly compare the effusivity of our phase change composites with standard, non-phase change materials, as is done in Fig. 3g. The same logic applies to Supplementary Fig. 11b.

$$e_\mathrm{app} = \frac{e_\mathrm{eff}}{\sqrt{2T_\mathrm{A}}}. \tag{16}$$

Supplementary Note 6:

Ultra-high thermal effusivity materials in thermal resonators – closed circuit experimental details.

The beneficial effects of ultra-high thermal effusivity materials towards thermal energy harvesting via thermal resonance devices, in terms of power output, are demonstrated experimentally in Fig. 3g. Details of the experimental setup are provided in Supplementary Fig. 9, as well as in the Methods and Supplementary Methods. Briefly, a temperature-controlled, programmable environmental stage provided the input, oscillating temperature boundary conditions. The input boundary conditions were monitored using two thin film K-type thermocouples on the outer surfaces of the thermal resonator. For Fig. 3g, the length of thermal mass 1 remained invariant (1.3 cm), while the material type was varied (Supplementary Fig. 10a and b). The length of the dominant thermal mass was chosen such that ν was sufficiently greater than 1. This results in a constant Q (value of 1/8) and a power density solely related to the thermal effusivity of the dominant thermal mass. Mathematical details are provided in Supplementary Note 10. A control experiment of this setup – in the absence of thermal masses 1 and 2 – is also provided in Supplementary Fig. 9 and shows negligible power generation, as anticipated.

As a comparison to the plot generated in Fig. 3g, we also generated plots of the average power output with respect to the thermal conductivity and heat capacities of the thermal masses in Fig. 3g, as shown in Supplementary Fig. 11. The results do not show an apparent trend in average power output with respect to either variable. As previously mentioned, the apparent heat capacity for the phase change materials was calculated with Supplementary Eq. (14).

Supplementary Note 7:

Converting thermal resonator power and voltage experimental data to a temperature difference.

The power profiles plotted for our thermal resonance devices (Fig. 3) are calculated using Supplementary Eq. (17) by measuring the closed circuit voltage over a 1.5 Ω resistor. Modeling the thermoelectric as a voltage source, the relationship between the closed circuit voltage and temperature difference is given by Supplementary Eq. (18).[20]

$$P = \frac{V^2}{R_{\mathrm{ext}}}. \tag{17}$$

$$V = \frac{\Gamma_{\mathrm{S}}\Delta T}{(1+R_{\mathrm{int}}/R_{\mathrm{ext}})}, \tag{18}$$

where Γ_{S} is the Seebeck coefficient, ΔT is the temperature difference, V is the voltage, R_{int} is the internal electrical resistance of the thermoelectric, and R_{ext} is the external electrical resistance in the circuit.[19] For the thermoelectrics considered in this section and the following section, we previously determined the internal resistance of the device to be 1.7 Ω using Supplementary Eq. (18) and considering the device to be a voltage source.[20] Supplementary Eqs. (17) and (18) can be used to back calculate the temperature difference existing across the central thermoelectric throughout the experiments using the external electrical resistance (1.5 Ω) and the Seebeck coefficient, as calculated below:

$$V_{\mathrm{OC}} = \Gamma_{\mathrm{S}}\Delta T, \tag{19}$$

where V_{OC} is the open circuit voltage.[19]

For our 1.5 cm x 1.5 cm thermoelectrics (Custom Thermoelectric; 03111-5L31-03CF), we applied a temperature difference of 16 °C (T_1 = 51 °C; T_2 = 35 °C) and measured an open circuit voltage of 84 mV. This yields an estimate of 5.2 mV K^{-1} for the Seebeck coefficient.

For converting open voltage circuit data (e.g., Supplementary Fig. 12) to a temperature difference, only Supplementary Eq. (19) is necessary.

Supplementary Note 8:

Comparison to pyroelectric energy harvesting.

The total energy (E) stored in a pyroelectric material at the end of a temperature variation (ΔT) is given by Supplementary Eq. (20).[21]

$$E = \frac{1}{2}\frac{p^2}{\varepsilon_{33}^{\sigma}} AL(\Delta T)^2, \quad (20)$$

where p is the pyroelectric coefficient, $\varepsilon_{33}^{\sigma}$ is the permittivity in the direction of polarization at constant stress, A is the area, and L is the length of the pyroelectric material. For a typical pyroelectric material – lead zirconate titantate (PZT) – the pyroelectric coefficient and permittivity are -380 µC m^{-2} K^{-1} and 2.6 x 10^{-9} C^2 J^{-1} m^{-1}, respectively.[21] Inserting dimensions identical to the resonators in Fig. 3g (A=1.5 cm x 1.5 cm) and L=1.3 cm, as well as considering the amplitude of temperature and time scale of temperature variations in Fig. 3e and 3f, we calculate an anticipated average power output of 250 µW for the PZT pyroelectric. It should be noted that this calculation is an upper bound on the energy generated by this pyroelectric material, as it only calculates the energy stored within the material at the end of the temperature change and does not consider the cycle by which it is discharged.

As a comparison to more optimized pyroelectric energy harvesting materials and techniques, we consider work from Kandilian et al.[22] The authors report to harvest 100 mJ cm^{-3} per cycle using a PMN-32PT single crystal capacitors. The thermal energy was harvested using the Olsen cycle and the temperature was varied at non-ambient conditions from 80 ºC to 170 ºC, a three times larger temperature change than for our closed circuit experiments. We estimate the average power output of their device - sized similarly to our devices and operating on a similar time scale – to be 490 µW.

Supplementary Note 9:

Thermal resonators – open circuit experimental details.

With the experimental setup shown in Supplementary Fig. 12a and b, the relationship between the thermal diffusion time scale of the dominant thermal mass and the ideal temperature oscillation frequency was also investigated, as shown in Supplementary Fig. 12c and d, to compare with the theoretical relationship shown in Fig. 5a. The experimental setup shown in Supplementary Fig. 12 was used to probe the open circuit performance of three thermal resonator designs over a broad range of temperature oscillation frequencies. Thermal mass 2 (steel, $L = 0.48\ \mathrm{cm}$) was identical for all device designs, while thermal mass 1 (Teflon) was varied in thickness (0.32 cm, red; 0.48 cm, blue; or 0.96 cm, green). All thermal masses have a 1.5 cm x 1.5 cm cross-section (Supplementary Methods). Based on the definition of v given in Eq. (10), we expect the ideal dimensional frequency for operation to be inversely related to the time scale of thermal diffusion for thermal mass 1, which is reflected by the experimental data. Furthermore, the experimental data were manually fitted to the performance factor model in Eq. (9), as shown by the color-corresponding solid lines. The shapes of the experimental data curves show resemblance to the fits obtained from the analytical model. The ideal frequency for thermal resonator operation, as shown in the experimental data, along with the results of Eq. (11), were used to calculate an average thermal diffusivity for the Teflon with a 95% confidence interval: $\alpha = (0.9 \pm 0.3) \times 10^{-7}\ \mathrm{m^2\ s^{-1}}$.

Experimental data for Supplementary Fig. 12d is fit to theory by first computing the average square value of the open circuit voltage, V_{avg}^2, for the thermoelectric heat engine, which is related to the performance factor through an effective Seebeck coefficient: $Q = \frac{\Delta T_{\mathrm{avg}}^2}{4T_{\mathrm{A}}^2} = \frac{V_{\mathrm{avg}}^2}{4\Gamma_{\mathrm{S}}^2 T_{\mathrm{A}}^2} = \frac{V_{\mathrm{avg}}^2}{\Gamma_{\mathrm{eff}}^2}$. This is computed for each temperature oscillation frequency. The frequency at which V_{avg}^2 is optimized (i.e., Q is optimized) is used to determine the thermal diffusivity of thermal mass 1, using the results from Eq. (11). The solid line corresponding to theory is then generated by inputting the fitted thermal diffusivity and the known length of thermal mass 1, as well as the reference thermal diffusivity value ($3.352 \times 10^{-6}\ \mathrm{m^2\ s^{-1}}$) and size for the invariant steel thermal mass 2. The experimental results were then scaled to the theory by adjusting the effective Seebeck coefficient.

Supplementary Note 10:

Theory: relationship between thermal effusivity and power output.

The derivations demonstrating the relationship between the estimated power output and the thermal effusivity for a particular thermal resonator design and environment are provided below. For an ideally-tuned thermal resonator, the power output is proportional to the thermal effusivity of the dominant (*j*=1) thermal mass.

For a generic heat engine between two thermal reservoirs with a cold temperature (T_C) and a hot temperature (T_H), the work (W) produced by the heat engine can be written as follows.

$$W = \eta\, q_H = \frac{\eta}{1-\eta} q_C, \tag{21}$$

where q_H is the heat flow from the hot reservoir to the heat engine and q_c is the heat flow delivered to the cold reservoir from the heat engine. η is the thermodynamic efficiency of the heat engine, which for a thermoelectric is given below.[19]

$$\eta = \left(1 - \frac{T_C}{T_H}\right) \frac{\sqrt{1+ZT}-1}{\sqrt{1+ZT}+T_C/T_H}\,, \tag{22}$$

where ZT refers to the figure of merit for the thermoelectric, which for typical commercial thermoelectrics is approximately 1. This corresponds to thermoelectric conversion efficiencies of approximately 1%. With this in mind Supplementary Eq. (21) can be simplified as follows.

$$W = \eta\, q_H \approx \eta\, q_C. \tag{23}$$

Given that thermal mass 1 limits the flux entering and exiting the heat engine, the maximum power achieved by the thermal resonator will be related to the maximum heat flux entering and exiting thermal mass 1 ($|J_{\max}|$). We can then write the maximum power density ($p_{\max}$) as follows.

$$p_{\max} = \eta\, |J_{\max}|\,. \tag{24}$$

An estimate of the time-averaged power density of the device is then given by:

$$p_{\text{avg}} \approx \eta\, |J_{\max}|\, Q. \tag{25}$$

Derivations for $|J_{\max}|$ are shown below. We first start with the temperature profile of thermal mass *j* with respect to space and time.

$$T_j(x_j, t) = T_0 + T_A \text{Re}[e^{i\omega t}] \frac{\text{Re}\left[\cosh\left(\sqrt{\frac{i\omega}{\alpha_j}}(L_j - x_j)\right)\right]}{\text{Re}\left[\cosh\left(\sqrt{\frac{i\omega}{\alpha_j}} L_j\right)\right]}\,. \tag{26}$$

With the heat flux entering and exiting thermal mass *j* being given by:

$$J_j = -k_j \left.\frac{\partial T_j}{\partial x_j}\right|_{x_j=0}. \tag{27}$$

Assuming $R \ll 1$, we focus on thermal mass j=1, and obtain the following flux entering and exiting the thermal mass.

$$J_1 = k_1 T_A \sqrt{\frac{i\omega}{\alpha_1}} e^{i\omega t} \tanh\left(\sqrt{\frac{i\omega}{\alpha_1}} L_1\right). \quad (28)$$

To obtain the maximum heat flux entering and exiting the thermal mass ($|J_{\max}|$), we multiply Supplementary Eq. (28) by its complex conjugate and evaluate the amplitude.

$$|J_{\max}|^2 = k_1^2 T_A^2 \frac{\omega}{\alpha_1}\left(\frac{e^{2b}+e^{-2b}-2\cos 2b}{e^{2b}+e^{-2b}+2\cos 2b}\right) = k_1^2 T_A^2 \frac{\omega}{\alpha_1}\left(1 - \frac{4\cos 2b}{2\cosh 2b + 2\cos 2b}\right), \quad (29)$$

where $b = L_1\sqrt{\frac{\omega}{2\alpha_1}}$.

Assuming $b \gg 1$, which is reasonable for our system, especially for an ideally-tuned resonator $\left(\frac{L_1^2}{\alpha_1}\omega = 10.8\right)$, we obtain

$$|J_{\max}| = k_1 T_A \sqrt{\frac{\omega}{\alpha_1}} = k_1 T_A \sqrt{\frac{\omega \rho_1 C_{p,1}}{k_1}} = \sqrt{k_1 \rho_1 C_{p,1}} T_A \sqrt{\omega} = e_1\, T_A\, \sqrt{\omega}\,, \quad (30)$$

$$p_{avg} \approx \eta\, |J_{\max}|\, Q = \eta\, e_1\, T_A\, \sqrt{\omega}\, Q. \quad (31)$$

With Supplementary Eq. (31) in mind, we can evaluate the linear fit in Fig. 3g to estimate the thermodynamic efficiency of the heat engine. Adjusting the units from the slope in Fig. 3g and accounting for the amplitude of temperature oscillation inputs ($T_A = 15$ °C) and the frequency of temperature oscillations ($\omega = 0.01\ \mathrm{s}^{-1}$), we estimate the efficiency of the thermoelectric heat engine as 0.3%. Note, we assume a value of 1/8 for Q, as discussed previously. From Supplementary Eq. (22), we calculate a maximum thermodynamic efficiency of a typical commercial thermoelectric (*ZT*=1) with our boundary conditions as 1.7%. An additional point-of-interest from Fig. 3g is the non-zero intercept shown by the experimental data (~50 μW). We postulate that this is due to heat leakage via natural convection of air at the interface of the bismuth telluride thermoelectric material and the ceramic plate to which it is adhered. An estimate for this intercept can be made according to Supplementary Eq. (32):

$$p_{avg} \approx \eta\, |J_{\max}|\, Q = \eta\, \bar{\bar{h}}\, T_A\, Q, \quad (32)$$

where $\bar{\bar{h}}$ is a heat transfer coefficient, which is approximately 10 W m^{-2} K^{-1} for natural convection in air.[23]

From Supplementary Eq. (32), we estimate the intercept to be 72 μW for a 1.7% efficient thermoelectric and a performance factor equal to 1/8 (assume planar area of thermoelectric 1.5 cm x 1.5 cm).

Supplementary Eq. (31) was also used to fit the data in Fig. 5b, which shows the development of a Ni/G/OD thermal resonator tuned to the dominant frequency mode of personal temperature fluctuations (Fig. 4b). The experimental data points were generated by varying the length of the dominant thermal mass (Supplementary Fig. 10c), simulating the temperature fluctuations using the experimental setup in Supplementary Fig. 9 and recording the average power output of the device. The following lengths were used for the Ni/G/OD thermal mass: 0.16 cm, 0.32 cm, 0.48 cm, 0.64 cm, 0.95 cm, 1.27 cm, and 1.91 cm. The average power with respect to time was calculated for each resonator and plotted vs. the square of the

length of the dominant thermal mass (experiment, red). The experimental data was fitted to the theory in Supplementary Eq. (31) using nonlinear minimization in MATLAB (fmincon). The fixed parameters for the model in Supplementary Eq. (31) were the efficiency of the heat engine (η =0.3% - from the fit in Fig. 3g), the amplitude of temperature fluctuations ($T_A = 15$ °C), and the frequency of temperature fluctuations ($\omega = 0.01\ s^{-1}$ – as given by the FFT in Supplementary Fig. 13. The parameters manipulated to obtain the fit were therefore k_1 and $\rho_1 C_{p,1}$. The following values for the thermal conductivity and volumetric heat capacity were obtained from the fit: $k_1 = 0.71\ \mathrm{W\ m^{-1}\ K^{-1}}$ and $\rho_1 C_{p,1} = 15\ \mathrm{MJ\ m^{-3}\ K^{-1}}$. These values correspond to an estimated apparent thermal diffusivity (4.7 x 10^{-8} m^2 s^{-1}) and effective thermal effusivity (17.9 J $cm^{-3/2}$ $(m\ s\ K)^{-1/2}$), which can be compared with calculations from Supplementary Eq. (13) (6.1 x 10^{-7} m^2 s^{-1}) and Eq. (2) (24.4 J $cm^{-3/2}$ $(m\ s\ K)^{-1/2}$ – also shown in Fig. 1c, respectively. Note, these values are calculated from our latent heat, thermal conductivity, and density measurements. The lower magnitudes of the apparent thermal diffusivity and effective thermal effusivity are likely attributed to the stacked nature of the Ni/G/OD composites and its creation of an in-series interfacial resistance.

Supplementary Note 11:

Diurnal thermal resonators from ultra-high thermal effusivity materials.

The heat map for diurnal thermal resonator design (Fig. 6a) was generated using Supplementary Eq. (31), assuming a thermoelectric efficiency of 1.1 %, a temperature oscillation frequency of $\omega = 7 \times 10^{-5}\ \mathrm{s}^{-1}$, and a temperature oscillation amplitude of $T_\mathrm{A} = 10\ ℃$, while incorporating k_1/L_1 and $\rho_1 C_{\mathrm{p},1} L_1$ as variables. The symbols for Ni/G/OD, OD, wet soil, and dry soil were generated as follows. The thermal conductivities (k_1) used in the calculations for the symbols in Fig. 6a for the PCM composites (OD and Ni/G/OD) were for the solid composite – assuming a negligible change in thermal conductivity with respect to phase. The thermal capacity was calculated for Fig. 6a for the PCM (OD and Ni/G/OD) with $\left(\frac{\rho h}{2T_\mathrm{A}}\right)$ from Supplementary Eq. (14). L_1 was calculated for the PCM composites using the results from Eq. (11) and assuming a thermal diffusivity (α_1) given by Supplementary Eq. (13). A standard heat capacity and solid thermal conductivity were used for the wet and dry soil calculations in Fig. 6a, with L_1 again given by results from Eq. (11).[24]

Detailed descriptions of the OD and Ni/G/OD diurnal resonators that were employed in a non-simulated environment are provided in Supplementary Fig. 14.

The internal resistance of the 4 cm x 4 cm thermoelectrics (TEG2-126LDT) is calculated to be 8 Ω using a similar procedure to the one explained above for the 1.5 cm x 1.5 cm thermoelectrics. Thus, the total internal resistance for the four thermoelectrics wired in series is estimated to be 32 Ω.

Supplementary Note 12:

Convection analysis for reduced to practice diurnal thermal resonators.

The analytical model and the thermal resonator experiments using a simulated temperature environment do not consider convection. However, for the devices reduced to practice, it can be imagined that the power generated with high thermal effusivity materials will eventually be limited by the convective heat transfer as the thermal effusivity continues to increase. The upper threshold for the power generation of a convection-limited diurnal resonator is a value of interest. The average power density for such a resonator could be described by Supplementary Eq. (33), where J_{max} is now related to convection instead of conduction. Supplementary Eq. (33) considers convection limitations by the aluminum heat fin shown in Fig. 6b.

$$p_{avg} \approx \eta\, |J_{max}|\, Q = \eta \frac{\bar{\bar{h}} A_t}{A} z_o T_A\, Q, \tag{33}$$

where A is the area of our heat engines (64 cm^2), A_t is the total heat transfer area of the heat sink, given by Supplementary Eq. (34), and z_o is the overall heat fin efficiency, given by Supplementary Eq. (37).[25]

$$A_t = N_{fin} A_{fin} + A_{fin,b}, \tag{34}$$

where N_{fin} is the number of heat fins (21), A_{fin} is the area of the heat fin, given by Supplementary Eq. (35), $A_{fin,b}$ is the area of the bare region between fins, given by Supplementary Eq. (36).

$$A_{fin} = (2L_{fin} + d_{fin})W_{fin}, \tag{35}$$

where L_{fin} is the length of the fin (2.6 cm), d_{fin} is the thickness of the fin (0.24 cm), and W_{fin} is the width of the fin (13.7 cm).

$$A_{fin,b} = d_b W_{fin}(N_{fin} - 1), \tag{36}$$

where d_b is the bare distance between fins (0.36 cm).

$$z_o = 1 - \frac{N_{fin} A_{fin}}{A_t}(1 - z_{fin}), \tag{37}$$

where z_{fin} is the heat transfer efficiency of an individual fin, given by Supplementary Eq. (38).

$$z_{fin} = \frac{\tanh(m_{fin} L_{fin,c})}{(m_{fin} L_{fin,c})}, \tag{38}$$

where m_{fin} and $L_{fin,c}$ are given by Supplementary Eqs. (39) and (40), respectively.

$$m_{fin} = \sqrt{\frac{2(W_{fin} + d_{fin})\bar{\bar{h}}}{W_{fin} d_{fin} k_{fin}}}, \tag{39}$$

where k_{fin} is the thermal conductivity of our aluminum fins (240 W m^{-1} K^{-1}).[16]

$$L_{fin,c} = L_{fin} + \frac{d_{fin}}{2}, \tag{40}$$

Plugging in the relevant values for our heat fin, assuming natural convection ($\bar{\bar{h}} = 10$ W m^{-2}K^{-1}), a heat engine efficiency of 1%, a temperature oscillation amplitude of 10 K, and $Q = 1/8$, we obtain an upper bound for the convection-limited power density of our device: 330 μW cm^{-2}.

Supplementary Methods:

Heat capacity measurements.

The heat capacity of the materials (Ni/G/OD and OD) was measured using DSC (DSC Q100 from TA Instruments, -10 ℃ → 50 ℃, 5 ℃ min^{-1}). For the reported values of heat capacity, each plot corresponds to 1 sample with a mass of approximately 10 mg. The values were calculated using a sapphire (Al_2O_3) calibration material.[26]

Raman spectroscopy.

Raman spectra for Cu, Cu/G, Ni, and Ni/G were obtained using a Horiba Jobin-Yvon Raman Microscope, with excitation at 532 nm.

Closed circuit thermal resonator experiments with environmental stage.

Closed circuit thermal resonator experiments were performed with the setup shown in Supplementary Fig. 9 (Supplementary Note 6). A U-shaped copper strip (thickness ~ 1 mm) is contacted with a temperature-controlled, programmable stage (Temperature Controlled Microscopic Stage from Linkam Scientific) using thermal paste to provide parallel oscillating temperature boundary conditions to a thermal resonance device. The temperature-controlled stage is programmed to oscillate between 0 ℃ and 50 ℃ at a scan rate of 10 ℃ min^{-1}). Thermal contact resistances limit the upper and lower input temperatures that the thermal resonance device achieves, refer to Fig. 3. The copper strip is also in direct contact with a thermoelectric (Custom Thermoelectric; 03111-5L31-03CF; 1.5 cm x 1.5 cm) on one side using thermal paste, and on the opposing side, the copper strip contacts thermal mass 1 using thermal paste. The output closed-circuit voltage of the thermal resonance device is monitored over a 1.5 Ω resistor (impedance matched to thermoelectric) using an oscilloscope (DrDAQ data acquisition board). A description and schematic of the thermal masses used in Figs. 3 and 5 are provided in Supplementary Note 6 and Supplementary Fig. 10.

Thermal effusivity of standard materials.

Thermal conductivity and volumetric heat capacity data were obtained via a standard Transient Plane Source (TPS 2500S) device with the settings shown below:

1-styrofoam $[k = 0.039\ \mathrm{W\ m^{-1}\ K^{-1}};\ \rho C_\mathrm{p} = 0.033\ \mathrm{MJ\ m^{-3}\ K^{-1}}]\ [P_0 = 10\ \mathrm{mW}; 40\ s; \mathrm{points}\ 24-98]$
2-neoprene foam $[k = 0.053\ \mathrm{W\ m^{-1}\ K^{-1}};\ \rho C_\mathrm{p} = 0.106\ \mathrm{MJ\ m^{-3}\ K^{-1}}]\ [P_0 = 15\ \mathrm{mW}; 40\ s; \mathrm{points}\ 24-98]$
3-wood $[k = 0.152\ \mathrm{W\ m^{-1}\ K^{-1}};\ \rho C_\mathrm{p} = 0.454\ \mathrm{MJ\ m^{-3}\ K^{-1}}]\ [P_0 = 60\ \mathrm{mW}; 40\ s; \mathrm{points}\ 32-98]$
4-PVC $[k = 0.231\ \mathrm{W\ m^{-1}\ K^{-1}};\ \rho C_\mathrm{p} = 1.406\ \mathrm{MJ\ m^{-3}\ K^{-1}}]\ [P_0 = 75\ \mathrm{mW}; 160\ s; \mathrm{points}\ 44-111]$
5-Teflon $[k = 0.310\ \mathrm{W\ m^{-1}\ K^{-1}};\ \rho C_\mathrm{p} = 1.717\ \mathrm{MJ\ m^{-3}\ K^{-1}}]\ [P_0 = 100\ \mathrm{mW}; 80\ s; \mathrm{points}\ 44-111]$
6-neoprene rubber $[k = 0.452\ \mathrm{W\ m^{-1}\ K^{-1}};\ \rho C_\mathrm{p} = 1.744\ \mathrm{MJ\ m^{-3}\ K^{-1}}]\ [P_0 = 150\ \mathrm{mW}; 80\ s; \mathrm{points}\ 32-98]$

Open circuit thermal resonator experiments with potentiostat.

The open circuit frequency response of simple thermal resonator designs was probed with the potentiostat setup shown in Supplementary Fig. 12 (Supplementary Note 9). The oscillatory temperature boundary

conditions, used to mimic ambient temperature fluctuations, for the devices were generated with Bi_2Te_3 commercial thermoelectrics (Custom Thermoelectric; 03111-5L31-03CF; 1.5 cm x 1.5 cm) wired in parallel to a potentiostat (Princeton Applied Instruments Model 273A). The potentiostat was programmed to operate in a continuous, oscillatory manner. The voltage scan rate (1 mV s^{-1} – 500 mV s^{-1}) and amplitude (-1 V to +1 V) dictated the frequency of temperature oscillations that occurred at the boundaries of the thermal resonator. The output open circuit voltage of the devices (over the central thermoelectric) is monitored using an oscilloscope (DrDAQ data acquisition board). The Teflon thermal masses (1) used in these experiments had a cross-section of 1.5 cm x 1.5 cm and varied in thickness according to the legend in Supplementary Fig. 12. The invariant, low thermal resistance mass (2) was steel with a cross-section of 1.5 cm x 1.5 cm and a thickness of 0.5 cm.

Supplementary References:

1. Zhang, H. et al. A capric–palmitic–stearic acid ternary eutectic mixture/expanded graphite composite phase change material for thermal energy storage. *Compos. Part A Appl. Sci. Manuf.* **87,** 138–145 (2016).

2. Zalba, B., Marín, J. M., Cabeza, L. F. & Mehling, H. Review on thermal energy storage with phase change: materials, heat transfer analysis and applications. *Appl. Therm. Eng.* **23,** 251–283 (2003).

3. Agyenim, F., Hewitt, N., Eames, P. & Smyth, M. A review of materials, heat transfer and phase change problem formulation for latent heat thermal energy storage systems (LHTESS). *Renew. Sustain. Energy Rev.* **14,** 615–628 (2010).

4. Li, X. et al. Phase change behavior of latent heat storage media based on calcium chloride hexahydrate composites containing strontium chloride hexahydrate and oxidation expandable graphite. *Appl. Therm. Eng.* **102,** 38–44 (2016).

5. Liu, C., Yuan, Y., Zhang, N., Cao, X. & Yang, X. A novel PCM of lauric–myristic–stearic acid/expanded graphite composite for thermal energy storage. *Mater. Lett.* **120,** 43–46 (2014).

6. Šarler, B. Stefan's work on solid-liquid phase changes. *Eng. Anal. Bound. Elem.* **16,** 83–92 (1995).

7. Stefan, J. Über einige Probleme der Theorie der Warmleitung. *Akad. Mat. Natur.* **98,** (1889).

8. M. J. Richardson, A. W. Woods, *Proc. R. Soc. London A Math. Phys. Eng. Sci.* **2008**, *464*, 1029.

9. Vélez, C., Khayet, M. & Ortiz de Zárate, J. M. Temperature-dependent thermal properties of solid/liquid phase change even-numbered n-alkanes: n-Hexadecane, n-octadecane and n-eicosane. *Appl. Energy* **143,** 383–394 (2015).

10. Gustafsson, S. E. Transient plane source techniques for thermal conductivity and thermal diffusivity measurements of solid materials. *Rev. Sci. Instrum.* **62,** (1991).

11. Xiao, X., Zhang, P. & Li, M. Preparation and thermal characterization of paraffin/metal foam composite phase change material. *Appl. Energy* **112,** 1357–1366 (2013).

12. Xia, L., Zhang, P. & Wang, R. Z. Preparation and thermal characterization of expanded graphite/paraffin composite phase change material. *Carbon N. Y.* **48,** 2538–2548 (2010).

13. Yang, X., Yuan, Y., Zhang, N., Cao, X. & Liu, C. Preparation and properties of myristic–palmitic–stearic acid/expanded graphite composites as phase change materials for energy storage. *Sol. Energy* **99,** 259–266 (2014).

14. Zarr, R. R. & Pintar, A. L. SRM 1453, Expanded Polystyrene Board, for Thermal Conductivity from 281 K to 313 K. *NIST Spec. Publ.* **260,** 175 (2012).

15. Gustavsson, M., Karawacki, E. & Gustafsson, S. E. Thermal conductivity, thermal diffusivity, and specific heat of thin samples from transient measurements with hot disk sensors. *Rev. Sci. Instrum.* **65,** (1994).

16. Shackelford, J. F., Han, Y-H., Kim, S., & Kwon, S-H. CRC Materials Science and Engineering Handbook, 4th Ed. (CRC Press, Taylor & Francis Group, 2015).

17. Huang, L., Zhu, P., Li, G. & Sun, R. Improved wetting behavior and thermal conductivity of the three-dimensional nickel foam/epoxy composites with graphene oxide as interfacial modifier. *Appl. Phys. A* **122,** 515 (2016).

18. Kumar, P. & Topin, F. Thermal conductivity correlations of open-cell foams: Extension of Hashin-Shtrikman model and introduction of effective solid phase tortuosity. *Int. J. Heat Mass Transf.* **92,** 539–549 (2016).

19. Paul, D. J. Thermoelectric Energy Harvesting. *ICT-Energy-Concepts Towar. Zero-Power Inf. Commun. Technol.* 49–78 (2014).

20. Wang, S. et al. Microscale solid-state thermal diodes enabling ambient temperature thermal circuits for energy applications. *Phys. Chem. Chem. Phys.* **19,** 13172–13181 (2017).

21. Bowen, C. R. et al. Pyroelectric materials and devices for energy harvesting applications. *Energy Environ. Sci.* **7,** 3836–3856 (2014).

22. Kandilian, R., Navid, A. & Pilon, L. The pyroelectric energy harvesting capabilities of PMN–PT near the morphotropic phase boundary. *Smart Mater. Struct.* **20,** 55020 (2011).

23. Kreith, F., Manglik, R. M. & Bohn, M. S. Principles of Heat Transfer. (Cengage Learning, Inc., 2011).

24. Sorour, M. M., Saleh, M. M. & Mahmoud, R. A. Thermal conductivity and diffusivity of soil. *Int. Commun. Heat Mass Transf.* **1990**, *17*, 189.

25. Ong, K. S., Tan, C. F., Lai, K. C. & Tan, K. H. Heat spreading and heat transfer coefficient with fin heat sink. *Appl. Therm. Eng.* **112,** 1638–1647 (2017).

26. DSC 2920 CE Differential Scanning Calorimeter Operator's Manual. (TA Instruments, Delaware, 1998).
